# Engineering resilient gene drives for sustainable malaria control by predicting, testing and overcoming target site resistance in *Anopheles gambiae*

**DOI:** 10.1371/journal.pbio.3003879

**Published:** 2026-07-06

**Authors:** Ioanna Morianou, Lee Phillimore, Bhavin S. Khatri, Louise Marston, Matthew Gribble, Austin Burt, Federica Bernardini, Andrew M. Hammond, Tony Nolan, Andrea Crisanti

**Affiliations:** 1 Department of Life Sciences, Imperial College London, London, United Kingdom; 2 The Francis Crick Institute, London, United Kingdom; 3 Department of Microbiology and Immunology, Johns Hopkins Bloomberg School of Public Health, Baltimore, Maryland, United States of America; 4 Biocentis Srl, Milan, Italy; 5 Department of Vector Biology, Liverpool School of Tropical Medicine, Liverpool, United Kingdom; Cornell University, UNITED STATES OF AMERICA

## Abstract

CRISPR-based gene drives are selfish genetic elements with the potential to spread through entire insect populations for sustainable vector control. Gene drives designed to disrupt the reproductive capacity of females can suppress laboratory populations of the malaria mosquito, *Anopheles gambiae*. However, any suppressive intervention will inevitably exert an evolutionary pressure for resistance, and the likelihood of resistance emerging at natural population scales remains poorly defined. Here, we present a pipeline to quantify the evolutionary space for resistance, enabling accelerated discovery, engineering, and testing of both natural and drive-induced variants that could reverse gene drive spread. We applied our approach to stress-test a best-in-class suppression gene drive that has evaded resistance in all laboratory-contained releases to date, known as Ag(QFS)1. We showed that previously undetected resistant alleles can arise at low frequency, including a novel type of partially resistant alleles that can perturb drive-invasion dynamics. Integrating experimentally derived resistance rates with population genetic modeling shows that single-target suppression drives are unlikely to be robust at natural mosquito population sizes, even at highly constrained loci. Here, we engineer and validate multiplexed gene drives in *Anopheles gambiae*, that target multiple conserved sites, actively removing resistant alleles. Our models predict that such gene drives could supress large natural mosquito populations in the field.

## Abstract 2

Gene drives for malaria control can be undermined by rare target-site resistance arising in mosquitoes, even when lab tests suggest robustness. This study reveals how rare resistance emerges and how multiplexed drives can overcome it, moving vector control forward.

## Introduction

Synthetic gene drives can be used to spread desirable traits through natural pest or disease vector populations for sustainable genetic control. The most advanced gene drives to date are based on CRISPR components, and they bias their inheritance by converting the germline of a gene drive heterozygote to homozygosity. Briefly, they comprise genes encoding a Cas9 and gRNA inserted within their own recognition site in a chromosome, so that their expression promotes cleavage of the wild-type chromosome followed by homology-directed repair (HDR) using the gene drive chromosome as a template—a process called homing (S1A Fig). These have been adapted for use in fruitflies [1], mice [2] and mosquitoes [3–6], and have been shown to spread autonomously from low initial frequency [7–9].

Gene drives can be programmed to reduce the breeding potential of a species by disrupting target genes essential for female reproduction [4,10]. This strategy of population suppression shows great promise for control of the African malaria mosquito, *Anopheles gambiae* [7–9].

A major technical obstacle in developing these systems is target site resistance—mutations in the target locus that prevent cleavage by the gene drive [11–13] (S1A Fig). These alleles can either exist due to standing variation in the field or can be induced by the gene drive when cleaved chromosomes are repaired by error-prone end-joining (EJ) rather than HDR. The extent to which these alleles can limit gene drive spread depends on their initial frequency in the natural target population, how often they are generated, and whether they confer a fitness cost [14–16]. Consequently, predicting the emergence and selection of resistance has become a central challenge in gene drive development.

Resistant variants are distinguished into two categories depending on whether they restore a functional copy of the target gene (**R1**) or not (**R2**) [11,17]. **R1** alleles block homing, conferring no fitness cost, and can therefore be subject to strong positive selection in the face of a gene drive, reversing its spread [11,13,17]. Conversely, **R2** alleles block homing but confer strong fitness costs, such as complete sterility or inviability in homozygosity [17,18]. Depending on their frequency, and the fitness of drive-carrying individuals, **R2** alleles can perturb gene drive invasion dynamics, and in some cases form a stable equilibrium that could reduce the magnitude of population suppression [19]. However, their effect on gene drive spread is substantially less severe than that of functional resistance (**R1**) and, in most cases, **R2** alleles are not expected to be a significant barrier to gene drive efficacy [11,17].

We previously developed a population suppression gene drive, targeting a female-specific exon of the highly conserved *doublesex (dsx)* gene in *An. gambiae* [7], impairing female development and achieving complete population suppression in laboratory cage experiments without detectable resistance [7,9]. This design combined germline-restricted Cas9 expression [17], with a functionally constrained target site [7,20], to minimize resistance formation (S1B Fig). However, natural mosquito populations are order of magnitudes larger than those tested in the laboratory, raising the possibility that even extremely rare resistant alleles may get generated and become selected in the field (S1C Fig). Thus, efforts to predict, pre-empt, and mitigate resistance prior to a first gene drive release are warranted.

Here, we develop an experimental framework to quantify the evolutionary space for resistance at a highly constrained suppression gene drive target site. By combining high-throughput mutagenesis, functional screening, and population genetic modeling, we estimate the rate at which functionally resistant alleles arise and translate this into predictions of gene drive performance across realistic population sizes. Applying this approach reveals that resistance can emerge against a best-in-class single-target suppression drive, motivating the design and testing of multiplexed gene drive architectures that target multiple conserved sites (S2 Fig). We show that such designs can actively remove resistant alleles, and based on modeling, are predicted to enable robust population suppression at natural scales.

Our framework serves as a paradigm for designing and testing the robustness of population suppression gene drives prior to release in the field.

## Results

To determine the evolutionary space for resistance to evolve at the *dsx* target site of the best-in-class suppression drive Ag(QFS)1 [7], we focused on two sources of variation: standing variation detected in the field; and drive-induced variation, created by end-joining repair following Cas9-induced cleavage. Our approach was then to recreate and test variants for their ability to block gene drive activity. We define variant alleles that do not block gene drive activity as **non-resistant** irrespective of whether they maintain gene function; variant alleles that maintain gene function and fully block gene drive activity as **R1** (as previously [11]); variant alleles that are non-functional and block gene drive activity as **R2** (as previously [11]); and lastly variant alleles that maintain gene function, but only partially block gene drive activity as **R3**.

### Standing variation among natural mosquito populations may harbor resistant alleles

In *An. gambiae* the best performing population suppression gene drive strain to date targets a site (T1) at the boundary between intron 4 and exon 5 of the *dsxF* isoform, chosen for its high level of sequence conservation across species in the *Anopheles* genus [21] (S3A Fig). Among wild-caught mosquitoes sequenced as part of the first release of the *Anopheles gambiae* 1000 genomes (Ag1000G) project [22], only a single SNP was previously reported at the T1 site (G → A, 2R:48714641). In vitro experiments suggested this SNP may still be susceptible to cleavage by the Cas9 present in the gene drive [7,23]. Nonetheless, anticipating the need for second-generation gene drives targeting multiple sites, we expanded our search to two additional, non-overlapping potential target sites on the female-specific exon 5 and examined standing variation at all three sites (T1, T2 and T3), using phase 3 Ag1000G data that covered an extended sample size of >2,700 African malaria mosquitoes, comprising samples of *An. gambiae*, *An. coluzzii* and *An. arabiensis* collected from 19 countries (S3A Fig) [22]*.*

Across T1-T3, 7 SNPs were identified, 6 of which appeared in only 2 or fewer individuals (S3B Fig). The only SNP at an appreciable frequency was the original G → A SNP found in T1, a finding that was also recently corroborated by another analysis [24]. This G → A SNP was present at a 1.3% overall allelic frequency across all collection sites, but reached an allelic frequency as high as 25.9% in Angola, where it appeared to be in Hardy-Weinberg equilibrium, meaning that it is likely under no or minimal negative selection (S3B and S4 Figs). Since this mutation might preserve *dsx* function, it was prioritised for resistance testing.

### Drive-induced EJ can be simulated in a high-throughput mutagenesis screen to identify putatively resistant alleles at *dsx*

End-joining repair of chromosomes cleaved by the gene drive nuclease can be a more important source of resistant mutations. Investigating such drive-induced resistance can be challenging, particularly in a strain like Ag(QFS)1, because the high rates of HDR in the germline leave very few cleavage events that are resolved by end-joining (EJ). Thus, insects carrying putatively resistant alleles are extremely rare. Using rates of homing and EJ measured for Ag(QFS)1 (S1 Data) [7], we estimate that 1.75% of the progeny of drive heterozygotes will carry an EJ mutation. Thus, fewer than 200 EJ events would be expected amongst 10,000 progeny of drive heterozygotes.

Previous research has revealed that, in the embryo, most cleavage events are repaired by end-joining. Consequently, maternally deposited nuclease can contribute to high rates of drive-resistant mutations being generated in the germline [3,4,11,17,25,26]; in some cases, over 70% of non-drive alleles in the offspring can carry an EJ mutation [17].

**Fig 1:**
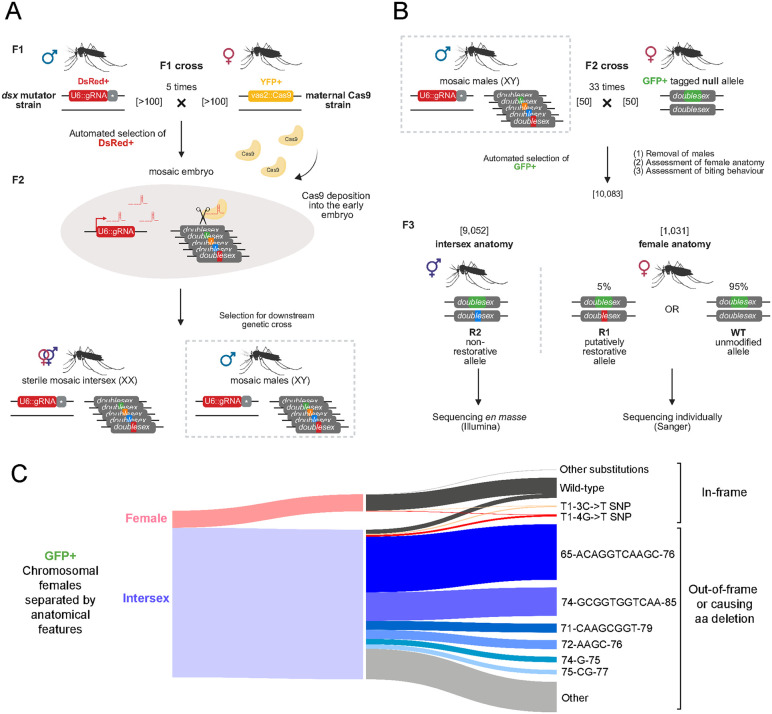
A high-throughput mutagenesis screen used to generate and assess Cas9-induced mutations revealed two putative resistant alleles at the target site of Ag(QFS)1. **(A)** A minimum of 100 males of a *dsx* mutator strain (DsRed^+^) expressing a ubiquitous gRNA against the Ag(QFS)1 target site and a linked *zpg*::*Cas9* (gray box), were crossed to a minimum of 100 females expressing *vas2*::*Cas9* (YFP^+^) (F1 cross, repeated 5 times). DsRed^+^YFP^*−*^ offspring were selected using the COPAS fluorescence-based larval sorter. Due to the high mutational load from deposited Cas9, females developed as mosaic intersex individuals that are sterile. Mosaic males were selected for a downstream genetic cross (F2) to detect and assess generated mutations. **(B)** F2 male DsRed^+^YFP^−^ offspring of the F1 cross were crossed to females containing a null *dsx-*F allele marked by GFP [7] (F2 cross). The GFP^+^ fraction of the offspring was selected using COPAS, to balance mutations inherited from males. Females with intersex anatomy must contain non-functional **R2** mutations, and females showing normal anatomy must contain putative functional **R1** mutations, or a WT unmodified allele. Anatomically intersex mosquitoes were analyzed *en masse* through pooled amplicon Illumina sequencing, and anatomical females were analyzed individually by Sanger sequencing. **(C)** The Sankey diagram shows the relative portion of GFP^+^ females that showed a female vs. intersex anatomy, and the relative proportion of the most common mutations discovered in them. The data underlying this figure can be found in S1 Data. Panels A and B were created in BioRender. Morianou, I. (2026) https://BioRender.com/u18x337.

We set out to simulate and enhance drive-induced EJ mutations at the target of Ag(QFS)1, and subsequently isolate the fraction of *dsx* alleles that may be resistant to gene drive activity. First, we created a new strain with the Ag(QFS)1 allele (comprising Cas9 and gRNA targeted to *dsx*) integrated away from its target site (“*dsx*-mutator” strain) so as to remove the possibility for homing. Next, to enhance EJ through maternal deposition, we crossed males of the *dsx*-mutator strain to females expressing Cas9 under the *vasa2* promoter (“maternal Cas9” strain) [3,17,25,26]. This approach generated high rates of mutagenesis in the F2, evidenced by close to 100% frequency of mosaic intersex in F2 females carrying the *dsx*-mutator allele and deposited Cas9 (Figs 1A, S5, and S6). However, high rates of intersex could be caused by just a few or even a single EJ event in early embryogenesis. To estimate of the minimum number of cleavage events occurring in the germline, we examined the progeny of 8 F2 males for the presence of distinct EJ alleles. We found that 96% of alleles were mutated, and that mutations were clustered with at least 1–4 different alleles in each clutch of 10 F3 sequenced individuals. These results are consistent with deposited nuclease activity occurring in the small number of stem-cell progenitors present during early embryonic development, prior to expansion of the germline (S7 Fig) [27].

To maximize the number of de novo EJ alleles we performed 33 crosses of 50 F2 males each (Fig 1B). To identify those alleles that may be resistant to gene drive activity, F2 males were crossed to an excess of females carrying a GFP*-*marked *dsxF*^*−*^ null allele. Since *dsxF* is haplosufficient and the GFP*-*marked allele is null, we could differentiate those individuals that received a non-functional R2 mutation, and therefore displayed intersex phenotype, from those that received either an unmodified wild-type (WT) allele or an **R1** mutation that restored DSX^F^ function, and exhibited normal female anatomy (Fig 1B). Assuming all 1,650 males gave progeny, and that distinct mutations were generated at the minimum rate of 2.75 per clutch (S7 Fig), we estimated that the present assay could have generated 4,538 de novo mutations.

Among approximately 20,000 F3 progeny carrying the GFP*-*marked *dsxF*^*−*^ null allele, that were examined as adults, 10,083 developed with non-male anatomy (i.e., female or intersex). The large majority (89.8%) of non-male F3 displayed the full intersex phenotype (S6 Fig), and pooled amplicon sequencing revealed that most of their alleles carried target site indels consistent with loss-of-function (Figs 1C, S8A, and S9), most of which had been previously described [7]. The remaining 10.2% of non-male F3 comprised females with apparently normal external morphology, of which most were able to take a blood meal (S1 Table). Anticipating that putative **R1** alleles could be enriched in these females, 852 were sequenced individually, revealing that only 3.6% (*n* = 31) carried an unambiguous target site modification, the majority being unmodified (Fig 1B and 1C; S1 Table). Four distinct alleles were identified in the group of modified anatomical females, all causing an amino acid substitution in the DSX^F^ protein. These putative R1 alleles include: a C → T substitution (2.2%) at position −3 of the T1 target site (2R:48714642, herein called T1-3C → T), a G → T substitution (1.1%) at position −4 of the T1 target site (2R:48714643, herein called T1-4G → T) and two alleles, containing more than one nucleotide substitution in the target site (0.4%) (Figs 1C, S8B, and S10; S1 Table). The two most common SNPs generated an alanine-to-valine and alanine-to-serine amino acid substitutions, respectively (S8B Fig).

We note that, occasionally, WT and putative R1 alleles were revealed at low frequency in the pooled sequencing of intersex individuals (Fig 1C). This may be explained by ongoing stochastic mosaicism caused by vestigial Cas9 activity (since *zpg*::*Cas9* is present in the *dsx*-mutator strain) in individuals that inherited the unmodified (WT) allele (see Materials and methods, S5A, S8A, and S9 Figs).

### Natural and Cas9-induced functional SNP variants confer varying levels of resistance to the Ag(QFS)1 gene drive

**Fig 2:**
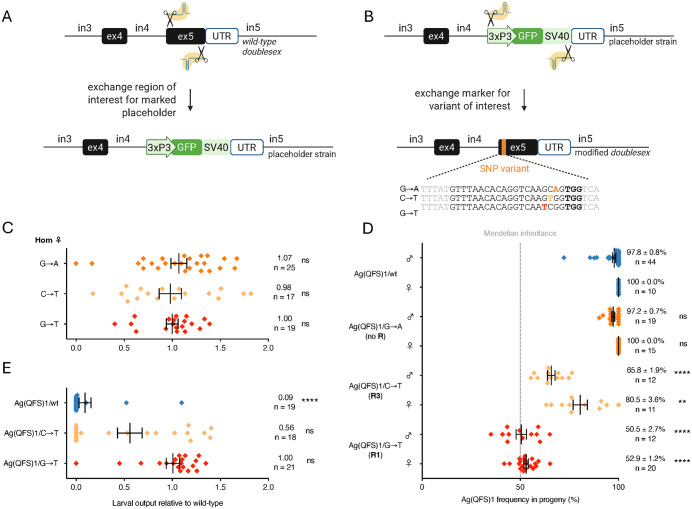
*De novo* engineering and testing of putative drive-resistant alleles revealed that each variant provided a different level of susceptibility to gene drive cleavage. **(A, B)** CRISPR-mediated cassette exchange (CriMCE) was used to engineer a naturally occurring (G → A, orange), and two Cas9-induced SNPs (C → T, light orange and G → T, red) at the T1 gene drive target site on *dsx* exon 5. **(A)** First, the whole exon 5 was exchanged for a GFP-marked placeholder. **(B)** The placeholder was then exchanged for a version of exon 5 containing one of the SNPs of interest. **(C)** The relative larval output of homozygous (Hom) females for each SNP, was found to be comparable to that of wild-type mosquitoes. Average larval output is shown to the right of each graph together with the sample size (*n*) and Welch’s *t* test statistical comparisons to each wild-type control (ns: non-significant, *p*-value = 0.5581, *η*^2^ = 0.0071, 95% CI: −0.1658 to 0.3035 for G → A; *p*-value = 0.8904, *η*^2^ = 0.0006, 95% CI: −0.3170 to 0.2765 for C → T; and *p*-value = 0.9955, *η*^2^ = 0.0000, 95% CI: −0.2069 to 0.2057 for G → T), after all datasets passed the D’Agostino–Pearson normality test. **(D)** Ag(QFS)1 frequency in the progeny of Ag(QFS)1/SNP *trans-*heterozygotes (Het) was compared to Ag(QFS)1/wt. Each SNP showed different propensity to cleavage by the gene drive. Mean Ag(QFS)1 transmission rates and the standard error around the mean (S.E.M.) are shown to the right of the graph, together with the sample size **(*n*)**. A Kruskall–Wallis non-parametric test was used, with Dunn’s post-hoc test to compare each group to the corresponding Ag(QFS)1/wt control (ns: non-significant, *p-*value > 0.9999 with 95% CI: −10.82 to 33.76 for Ag(QFS)1/G→A males and 95% CI: −33.15 to 33.15 for Ag(QFS)1/G→A females; **: significant, *p-*value = 0.0048 with 95% CI: −93.84 to −22.88 for Ag(QFS)1/C→T females, ****: significant, *p-*value < 0.0001 with 95% CI: −84.73 to −31.85 for Ag(QFS)1/C→T males, 95% CI: 53.35 to 106.23 for Ag(QFS)1/G→T males and 95% CI: 63.85 to 126.75 for Ag(QFS)1/G→T females). **(E)** The fitness cost in drive-heterozygote females was reduced or completely restored when Ag(QFS)1 was paired to a cut-resistant allele. The relative larval output of Ag(QFS)1/SNP and Ag(QFS)1/wt Het females was compared to a wild-type control. Mean relative larval output is shown to the right of each graph, together with the sample size **(*n*)**. A Kruskall–Wallis non-parametric test was used, with Dunn’s post-hoc test to compare each group to the corresponding wild-type control (****: significant, *p-*value < 0.0001, 95% CI: 11.04 to 68.24 for Ag(QFS)1/wt; ns: non-significant, *p-*value = 0.1566, 95% CI: −10.23 to 46.35 for Ag(QFS)1/C → T; and *p-*value > 0.9999, 95% CI: −24.03 to 27.33 for Ag(QFS)1/G → T). The data underlying this figure can be found in S7 Data, and the corresponding statistical analyses in S5 Data. Panels A and B were created in BioRender. Morianou, I. (2026) https://BioRender.com/uuksd4q.

Having identified both standing and drive-induced variants that are apparently functional, we next wanted to determine whether these variants confer resistance to drive activity and therefore represent bona fide **R1** alleles. Therefore, we engineered de novo the most frequent natural variant (T1-2G → A at position 2R:48714641 in the genome, S4 Fig), and the two most common putative **R1** variants identified in our mutagenesis screen (T1-3C → T and T1-4G → T, S8 Fig) to confirm that they retain functionality and test whether they also confer resistance to the Ag(QFS)1 gene drive. The relevant nucleotide changes were precisely inserted into the genome using CRISPR-mediated cassette exchange (CriMCE), which allows the efficient recovery of defined mutations generated by HDR, even in the absence of a selectable marker (Fig 2A and 2B) [28].

By performing blinded fertility assays we determined that females homozygous for each of the three engineered alleles are as fertile as wild-type (*p-*value = 0.5581 for G → A, *p-*value = 0.8904 for C → T and *p-*value = 0.9955 for G → T) (Figs 2C and S11). In homing assays, the T1-2G → A natural SNP was cleaved efficiently and did not reduce inheritance rates of the drive element in either sex (*p-*value > 0.9999) (Fig 2D), and therefore does not represent a resistant allele, confirming our previous in vitro findings [7]. In contrast, the T1-4G → T SNP was found to be an **R1** allele that completely blocks the bias in gene drive inheritance rates in both sexes (*p-*value < 0.0001) (Fig 2D). Interestingly, the T1-3C → T SNP did not block homing entirely, but significantly reduced drive transmission (65.8% inheritance in male carriers, *p-*value < 0.0001; 80.5% inheritance in female carriers, *p-*value < 0.0048) (Fig 2D). To our knowledge, this is the first description of a mutation that partially blocks gene drive activity, whilst maintaining gene function. We therefore termed this type of resistance **R3**.

Interestingly, the availability of the **R1** allele allowed us to confirm a previous hypothesis that reduced fertility in female carriers heterozygous for the drive (Ag(QFS)1/wt) was due to ‘leaky’ Cas9 expression in the soma causing mutations in the WT allele [7,9,29]. Females heterozygous for the drive and the **R1** allele (Ag(QFS)1/G→T) showed fertility equivalent to wild-type mosquitoes (*p-*value > 0.9999), while those with the partially cleavable **R3** allele (Ag(QFS)1/C→T) showed somewhat reduced fertility, though not significantly different from wild-type (*p-*value = 0.1566) (Fig 2E).

### The presence of partial resistance (R3) allows gene drive invasion but prevents population elimination

**Fig 3:**
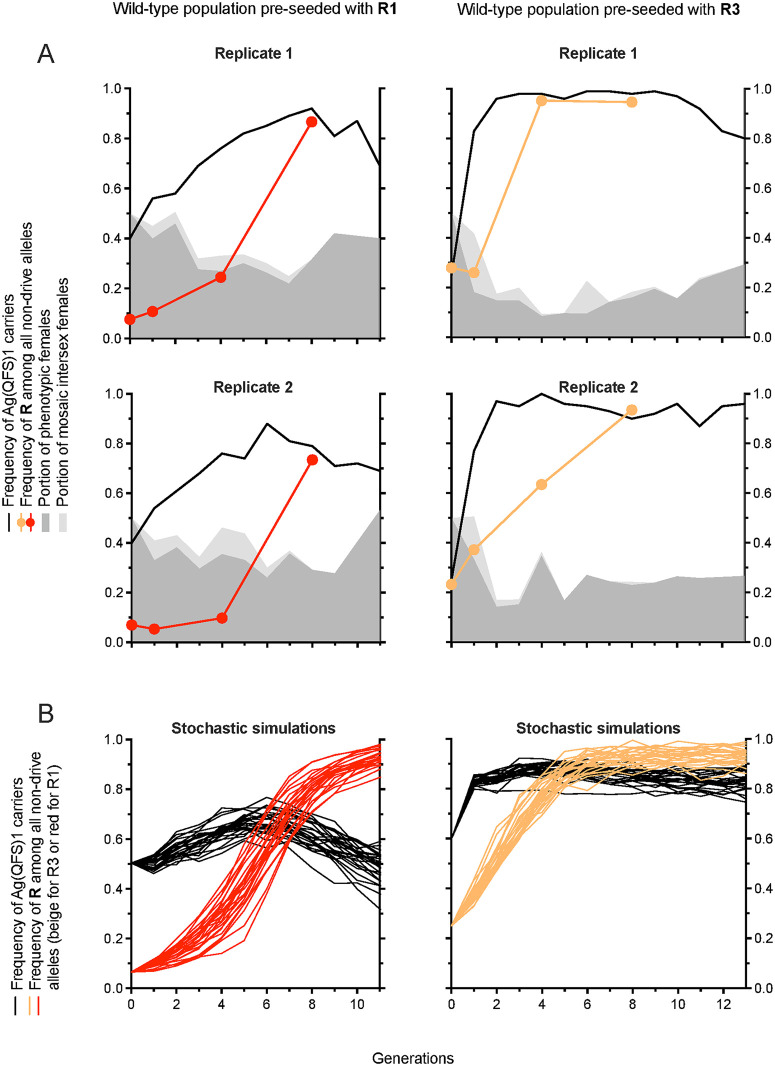
In the presence of R1 resistance Ag(QFS)1 spread is reversed, whereas in the presence of R3 resistance Ag(QFS)1 spreads, maintaining a high frequency, but ultimately fails to eliminate caged populations. **(A)** Ag(QFS)1 heterozygous males were released at a frequency of 40% (R1 cages, left) and 25% (R3 cages, right) in duplicate populations pre-seeded with 15% R1 (left) and 25% R3 (right) resistant alleles, respectively. The frequency of Ag(QFS)1 carriers (solid black line) and resistant alleles amongst all non-drive alleles (red for R1 and light orange for R3, circles denote sampled generations) was tracked over discrete generations using pooled amplicon sequencing. The proportion of females that developed normally as pupae (dark gray shaded region) or abnormally (mosaic intersex, light gray shaded region) is also shown. Note that fully intersex females and males are indistinguishable at the pupal stage from one another. **(B)** Stochastic simulations of caged populations pre-seeded with R1 (left) or R3 (right) resistance, using a fixed population size of 600 mosquitoes. Solid black lines refer to the frequency of Ag(QFS)1 carriers, and color lines to the frequency of resistant alleles among all non-drive alleles (red for R1, light orange for R3). The data underlying this Figure can be found in S3 Data and https://doi.org/10.5281/zenodo.20558598.

The litmus test of resistant alleles is their ability to be selected in a population and compromise the ability of a gene drive to invade. Starkly different population outcomes are possible depending on the type of resistance and the design of the gene drive itself [11,12,17,19]. Particularly interesting is the potential impact of **R3** alleles, which have not been described previously. Here, we introduced Ag(QFS)1 into caged populations pre-seeded with **R1** or **R3** resistance and tracked the frequency of resistant and drive alleles over time, together with the fraction of putatively fertile females as a measure of population suppression (Fig 3A and 3B).

In cages seeded with both gene drive heterozygote males (at an overall frequency of 40%) and the **R1** allele (T1-4G → T) at a frequency of 9%, the **R1** allele increased rapidly within 8 generations, to 74%–87% of all non-drive alleles in replicate cages (Fig 3A). In both replicates, as the **R1** allele began to reach high frequency the number of gene drive carriers started to decrease, in line with our predictions based on stochastic simulations (Fig 3B), and mirroring the dynamics observed for **R1** alleles at other gene drive target sites [11,17]. As expected, in the presence of the **R1** allele there was little overall population suppression caused by the drive.

In cages seeded with gene drive heterozygote males (at an overall frequency of 25%) and the **R3** allele (T1-3C → T) at a frequency of 25%, the observed invasion dynamics were markedly different, and unique for a population suppression gene drive: near-full invasion of the drive did not cause complete population elimination, but rather a sustained ~40% drop in reproductive output resulting from a reduction in the fraction of phenotypically normal females (Fig 3A). This outcome is explained by the rapid selection of the **R3** allele that replaces the wild-type allele, resulting in an effectively weaker drive unable to eliminate the population—an outcome that is also predicted in stochastic modeling (Fig 3B).

**Fig 4:**
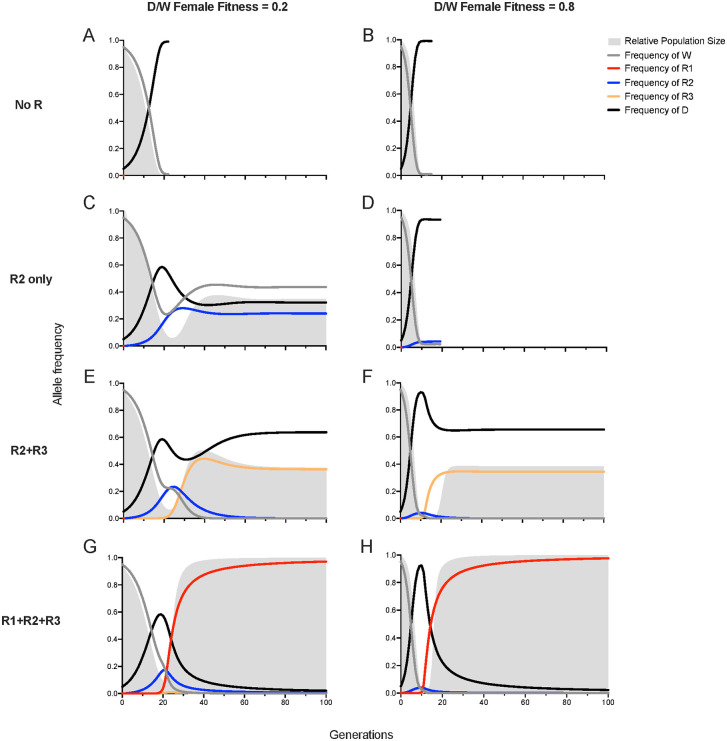
Effectively deterministic simulations of gene drive spread in the presence of no resistance, or R1, R2 and R3 resistance. Generation 0 of each simulation assumes a 0.05 drive (D) allele frequency brought about from a 10% release of male gene drive heterozygotes (D/W). Simulations were run on a panmictic population size of 10^12^, assuming no standing variation. All simulations assume full fitness for male gene drive heterozygotes (D/W), but reduced fitness for female gene drive carriers (D/W) of either 0.2 **(A, C, E, G)** or 0.8 **(B, D, F, H)** fitness relative to wild-type. The gray shaded region represents the total population size relative to the starting population of 10^12^. The solid lines represent frequency of wild-type (W) in gray, gene drive (D) in black, R1 in red, R2 in blue and R3 alleles in light orange. In the “no resistance” simulations (A, B) the rate of wild-type allele conversion to an end-joining allele was set to 0. Based on Ag(QFS)1 gene drive transmission data, for the rest of the simulations (C, H) the rate of wild-type allele conversion to an end-joining allele (whether R1, R2 or R3) was set to 0.035. For “R2 only” simulations (C, D) the rate of R2 allele creation out of all EJ alleles was set to 1, whilst R1 and R3 were set to 0. For “R2+R3” simulations (E, F) the rate of R2 allele creation out of all EJ alleles was set to 0.9975, and R3 was set to 0.0025. For “R1+R2+R3” simulations (G, H) the rate of R1 allele creation out of all EJ alleles was set to 0.0016, R2 was set to 0.9959, and R3 was set to 0.0025. The data underlying this Figure can be found in S6 Data.

To understand the potential impact of **R1**, **R2** and **R3** alleles, we simulated the dynamics of drive invasion and population suppression under different scenarios (Fig 4), assuming rates of allele creation determined in the mutagenesis screen (S2 Table).

First, assuming no resistance, modeling simulations predict a rapid population elimination when the fitness of drive heterozygous females is high (fitness = 0.8), and similar but delayed dynamics when fitness is low (fitness = 0.2) (Fig 4A and 4B).

Second, assuming only non-functional **R2** resistant mutations arise, we observe rapid population elimination when the fitness of drive heterozygous females is high, but a stable suppression of ~65% without elimination when fitness is low (Fig 4C and 4D). These results agree with previous findings [19], supporting the hypothesis that population-level outcomes depend on the fitness of gene drive carriers.

Third, assuming partially resistant **R3** alleles arise in addition to **R2**, we predict a stable suppression without elimination (Fig 4E and 4F). In contrast to **R2**, the dynamics of spread and eventual stabilization of **R3** are almost identical for both low and high fitness of drive heterozygous females, wherein **R3** eventually displace both **R2** and WT alleles. The extent of population suppression will depend upon the fitness and homing-blocking activity of the specific **R3** allele, which can be expected to vary greatly for different gene drive designs and resistant alleles. Assuming the characteristics of the **R3** allele discovered in this study, we predict a stable suppression of ~60% (Fig 4E and 4F).

Finally, assuming all three types of resistant alleles arise (**R1**, **R2** and **R3**), we predict that the **R1** allele will become fixed in the population, displacing all other alleles including the gene drive, and allowing the population to recover in size (Fig 4G and 4H). These results are consistent with previous experimental and theoretical investigation of **R1** and **R2** resistance [11,17,19].

### Multiplexed gene drives could mitigate resistance in natural populations

It has long been recognized that robust gene drive design should incorporate redundancy by targeting multiple sites simultaneously [10,14,25]. To understand the potential effectiveness of this approach, we sought to estimate the rate of resistance arising at one target and extrapolate to a multiplexed gene drive assuming equal rates at all three sites.

**Fig 5:**
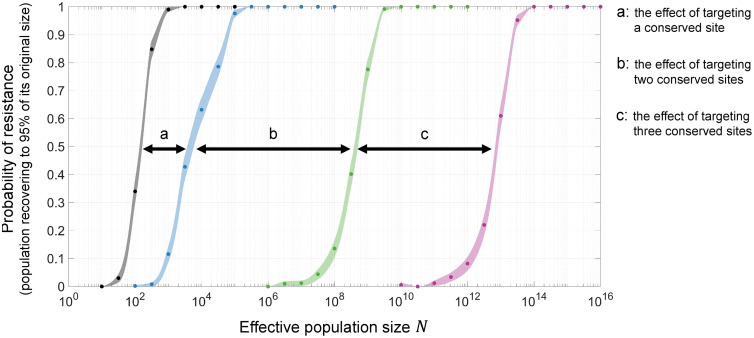
Simulations calculating the probability of resistance evolving against a gene drive targeting a single or multiple sites, given an increasing target population size. The probability of resistance decreases with targeting a conserved site, and with each subsequent conserved site targeted. Each data point is the average of 500 replicate stochastic simulations at the population size shown with 95% confidence intervals represented by the shaded regions, which have been smoothed for visual clarity*.* The model assumes no standing variation and a rate of wild-type allele conversion to an end-joining allele of 0.035 for all simulations*.* Simulations in gray assume a single gene drive target site with a rate of resistant allele creation of 1/3 amongst end-joining alleles. Simulations in blue assume a single conserved gene drive target site, equivalent to the target site of Ag(QFS)1, with a rate of resistant allele creation as indicated by our mutagenesis screen, i.e., 0.0016 (for R1) + 0.0025 (for R3) = 0.0041 amongst end-joining alleles. Simulations in green and violet assume the same rate of resistant allele creation for each target site, but for two or three target sites in total, respectively. For all simulations, gene drive fitness was assumed to be the same as calculated for Ag(QFS)1. Resistance is defined by the population recovering to 95% of its original (wild-type) size. The data underlying this figure can be found in S9 Data.

Through our mutagenesis screen, we were able to estimate the rate (*β* or *γ*) at which either fully resistant (**R1**, *β* = 0.0016) or partially resistant (**R3**, *γ* = 0.0025) mutations might arise by EJ repair. In turn, using our stochastic modeling framework, we incorporated these values to calculate the mean probability of resistance arising in populations of different sizes (Fig 5). For comparison, we also calculated the probability of resistance arising at target sites with minimal sequence conservation, where a third of indels preserve the reading frame (Fig 5).

We find that the probability of resistance increases monotonically as the population size increases, as $p_{R}(N)\approx \text{1}-e^{-N/N^{*}}$where $N^{*}$ is the critical population size at which the probability of resistance is ≈0.63 and is roughly given by N×β~1 [16], where β is the rate of generation of **R1** alleles. By numerically finding the population size for which $p_{R}(N)=\text{0}.\text{0}\text{5}$, we can define a maximum critical population size, $N_{max}$, below which we can guarantee the prevention of resistance to at least 95% confidence. We observe that increasing the conservation of a site from β=1/3 to β=0.0016, increases $N_{max}\;$from 38 to 599 individuals (Fig 5). This is similar to the population size of existing population invasion experiments testing Ag(QFS)1 spread in small and large cages [7,9]. Indeed, simulations of laboratory experiments indicated that de novo generation of **R1** and **R3** is rare, with **R1** being expected to arise in 0.5% and **R3** in 1.9% of replicate cage experiments. Further, we predict that using 2 gRNAs would give protection up to $N_{max}\approx \text{3}.\text{6}\times {\text{1}\text{0}}^{\text{7}}$ individuals, whilst 3 gRNAs would give protection up to $N_{max}\approx \text{5}.\text{2}\times {\text{1}\text{0}}^{\text{1}\text{1}}$ individuals.

Estimates of natural mosquito population sizes vary between ${\approx \text{1}\text{0}}^{\text{6}}\;$(based on nucleotide diversity [23]), which is likely downward biased due to historical bottlenecks; to ${\approx \text{1}\text{0}}^{\text{7}}$ (based on demographic history inference methods [30,31]); and up to $\approx \text{2}\times {\text{1}\text{0}}^{\text{8}}\;$(based on the analysis of a recent soft sweep of insecticide resistance alleles [32], updated to employ more recent estimates of the nucleotide mutation rate in *An. coluzzii* ($\mu \approx {\text{1}\text{0}}^{-\text{9}}$) [33]). On this basis we would expect that 3 gRNAs targeting the female-specific exon of *dsx* (S3A Fig), would provide sufficient protection against the evolution of resistance.

### Multiplexing at the *dsx* locus improved drive transmission dynamics and mitigated resistance by actively removing resistant alleles

**Fig 6:**
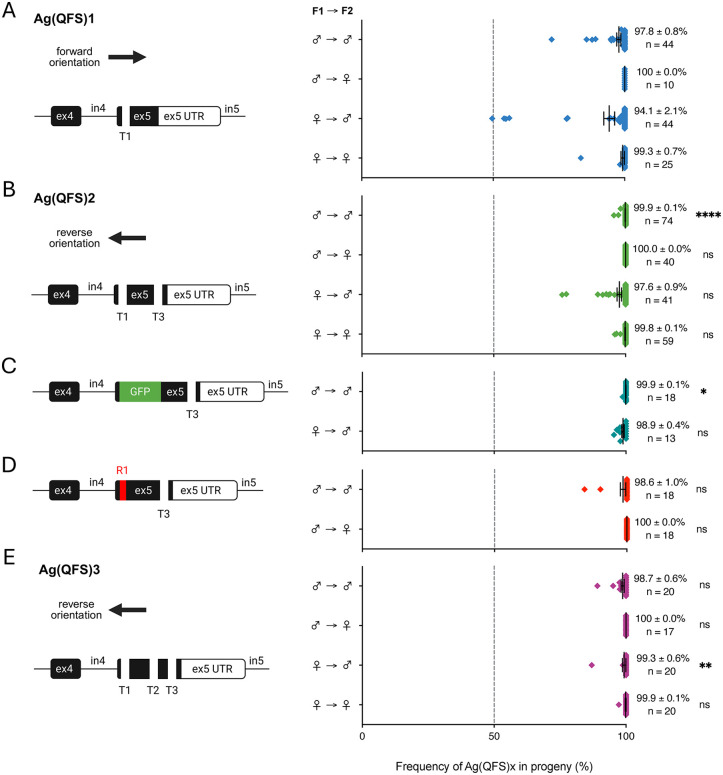
Transmission rates of multiplexed gene drives Ag(QFS)2 and Ag(QFS)3 in comparison to Ag(QFS)1. Multiplexed gene drives showed equivalent or improved transmission rates compared to Ag(QFS)1, and Ag(QFS)2 retained efficient drive bias when T1 was blocked by an R1 allele or a GFP insertion. Schematics to the left show the gene drive tested, its orientation, and the number and location of available cut sites in each experimental set-up. Graphs to the right show the gene drive frequency in the progeny of gene drive heterozygotes crossed to wild-type: **(A)** Ag(QFS)1/wt (blue); **(B)** Ag(QFS)2/wt (green); **(C)** Ag(QFS)2/*dsxF*^*−*^ (teal); **(D)** Ag(QFS)2/R1 (red); **(E)** Ag(QFS)3/wt (violet). Sex notation to the left of each graph indicates the sex of the gene-drive carrying grandparent and parent of the scored progeny, respectively (e.g., M→F: offspring of female gene drive carriers crossed to wild-type males that inherited the drive from a *male carrier*). Mean gene drive transmission rates and the standard error around the mean (S.E.M.) are shown to the right of each graph, together with the sample size (*n*) and Kruskall–Wallis statistical comparisons to the Ag(QFS)1/wt control (blue, panel **A)**: **(B)** for Ag(QFS)2/wt, ****: significant with *p-*value < 0.0001, 95% CI: 63.32 to 94.46 for M→M; ns: not significant with *p-*value > 0.9999, 95% CI: −28.71 to 28.71 for M→F, *p-*value > 0.9999, 95% CI: 11.23 to 46.67 for F→M, and *p-*value > 0.9999, 95% CI: −11.80 to 26.95 for F→F. **(C)** for Ag(QFS)2/*dsxF*^*−*^, *: significant with *p-*value = 0.0206, 95% CI: 54.83 to 100.41 for M→M, ns: not significant with *p-*value > 0.9999, 95% CI: −10.59 to 40.81 for F→M; **(D)** for Ag(QFS)2/R1, ns: not significant with *p-*value = 0.1803, 95% CI: 37.41 to 82.99 for M→M, and *p-*value > 0.9999, 95% CI: −32.02 to 32.02 for M→F; **(E)** for Ag(QFS)3/wt, ns: not significant with *p-*value > 0.9999, 95% CI: −15.62 to 28.33 for M→M, *p-*value > 0.9999, 95% CI: −32.36 to 32.36 for M→F and *p-*value > 0.9999, 95% CI: −16.49 to 32.22 for F→F; **: significant with *p-*value = 0.0057, 95% CI: 61.37 to 105.33 for F→M. The data underlying this Figure can be found in S8 Data, and the corresponding statistical analyses in S5 Data. Schematics to the left of each graph were created in BioRender. Morianou, I. (2026) https://BioRender.com/gx187hy.

Based on our estimates of the perdurance of drives targeting multiple sites, we designed two new multiplexed gene drives to mitigate against all types of putative resistant alleles at the Ag(QFS)1 target site (T1). We developed two strains, targeting two (T1 and T3) or three sites on *dsx* exon 5 (T1, T2 and T3) (S3 Fig), and named them Ag(QFS)2 and Ag(QFS)3, respectively (S12 Fig). Both Ag(QFS)2 and Ag(QFS)3 gene drives showed high transmission rates that were comparable to or higher than those of Ag(QFS)1 when targeting a WT locus (Fig 6A, 6B, and 6E). This may be explained by overall higher cutting and repair at *dsx* in the presence of multiple gRNAs [25].

A fundamental tenet in employing a multiplexed gene drive is that it should be able to home at a target locus containing at least one susceptible site, even if other target sites within that locus have evolved resistance. Importantly, our multiplexed drives are designed to force resection of any resistant alleles, during homing, as long as one of the targets remains cleavable (S2 Fig).

We therefore tested whether Ag(QFS)2 could bias its inheritance when faced with the **R1** allele (T1-4G → T) at the T1 gene drive target site. Ag(QFS)2 showed high transmission rates (98.6%–100%) utilizing exclusively the T3 site available for cleavage (Fig 6D). In this case, a minimum of 76 nucleotides must be resected, past the **R1** allele, to generate ends with immediate homology to the drive allele to permit homing. In a separate experiment, forcing a resection of >1.2 kb, necessary to remove a dominant marker adjacent to a susceptible site, caused no detectable reduction in homing (Fig 6C). We note that under some circumstances, particularly where significant resection is required, homing and meiotic drive (removal of cleaved chromosomes) can co-occur [34–37]. Both processes result in removal of single resistant alleles and have broadly similar outcomes on gene drive dynamics. Together, these results suggest that there is ample width of sequence in which multiple sites could be chosen for a multiplexed gene drive, without affecting drive efficacy.

### Multiplexed gene drive carriers show marked improvements in fertility

**Fig 7:**
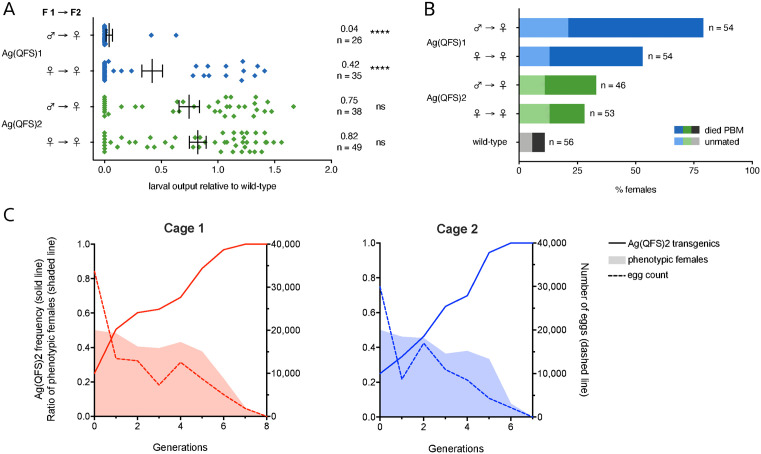
Female fitness and cage invasion of Ag(QFS)2 gene drive heterozygotes. The improved female fitness of Ag(QFS)2 multiplexed gene drive carriers contributed to efficient population invasion of small laboratory cages. **(A)** Relative larval output of Ag(QFS)1 and Ag(QFS)2 heterozygous females compared to wild-type (WT), distinguishing between paternal (M→F) or maternal (F→F) inheritance of the drive allele. Raw egg and larval output is shown in S16 Fig. The data are not normally distributed (D’Agostino–Pearson normality test) and were therefore compared to the WT control using the Kruskall–Wallis non-parametric test and Dunn’s post-hoc test for multiple comparisons: ****: significant with *p*-value < 0.0001, 95% CI: −114.34 to −75.58 for Ag(QFS)1 M→F and *p*-value < 0.0001, 95% CI: −76.11 to −40.87 for Ag(QFS)1 F→F; ns: not significant with *p*-value = 0.1270, 95% CI: −43.44 to −9.06 for Ag(QFS)2 M→F and *p*-value = 0.5570, 95% CI: −32.86 to −0.82 for Ag(QFS)2 F→F. **(B)** Survival and mating status of heterozygous females 3–4 days post-bloodmeal (PBM), compared to WT. Unmated females are depicted in pale colors and females that died PBM in solid colors. **(C)** Population invasion dynamics of Ag(QFS)2 in two replicate cages, following release of heterozygous males (M→M) into a wild-type population at an initial heterozygote frequency of 25% (12.5% allelic frequency). The total population size was maintained at approximately 600 adults by sampling up to 650 eggs each generation. The proportion of individuals carrying the Ag(QFS)2 allele (solid lines) was tracked over time by scoring the DsRed fluorescence marker, which distinguishes carriers regardless of zygosity, alongside the proportion of phenotypic females (shaded regions) and egg output per cage per generation (dashed line). The data underlying panels A and B can be found in S8 Data, and the corresponding statistical analyses in S5 Data. The data underlying panel C can be found in S4 Data.

Ag(QFS)2 heterozygotes showed equivalent female fitness (egg output, larval output, and egg hatching rate) compared to wild-type (S13 Fig), similar rates of survival from the larval stage into adulthood (S14 Fig), and equivalent male mating competitiveness (S15 Fig). Surprisingly, Ag(QFS)2 female carriers also showed higher general fertility and reduced somatic mosaicism, compared to the original Ag(QFS)1 strain (Figs 7A and S16). We observed that mosaicism was higher in females inheriting the drive element paternally, rather than maternally, as we did for Ag(QFS)1 [7,8], although the effect was less pronounced (Figs 7A, 7B, and S16). We also observed increased post-bloodmeal mortality in Ag(QFS)1 heterozygous females, a phenotype that was also present, albeit less severe, in Ag(QFS)2 heterozygotes (Fig 7B). Since blood-feeding is a female-specific trait in mosquitoes, it is perhaps not surprising that components relating to midgut metabolism might be controlled by DSX^F^, leading to increased post-bloodmeal mortality in mosaic individuals.

In testing multiplexed Ag(QFS)3 strains, we noticed that the level of somatic mosaicism of female gene drive carriers differed markedly according to the orientation of the gene drive element integrated within the *dsx* target gene. When the transcriptional units controlling the Cas9 and gRNAs were integrated in the same direction as *dsx* (‘forward’), the levels of somatic mosaicism were an order of magnitude higher than when the transgenes had integrated in the opposite (‘reverse’) direction (74.9 ± 13.9% compared to 7.6 ± 4.0%, respectively with *p-*value = 0.0104) (S17 Fig). This effect was reproducible across Ag(QFS)3 strains, each containing the same transgene, but generated independently from five different founders.

In light of this observation, the improved performance of the multiplexed Ag(QFS)2 strain over the single guide Ag(QFS)1 may be attributable, at least in part, to the orientation of the drive element, the former being integrated in the reverse, and the latter in the forward orientation (S17 Fig). Whether the effect of orientation is a direct consequence of the direction of transcription of the Cas9 or gRNAs relative to *dsx*, or of another feature related to the distinct gene drive architecture resulting from the two integration modes, is still unclear.

### Multiplexed gene drives targeting *dsx* can rapidly invade and supress laboratory populations

Taken together, multiplexed gene drive strains would be expected to show more efficient population invasion dynamics, even in the absence of any resistance. We therefore tested Ag(QFS)2 population invasion in laboratory cages, in a setup that mirrored the Ag(QFS)1 cage trials, using a release ratio of 1:1 heterozygous Ag(QFS)2 males to WT males (overall gene drive allele frequency of 12.5%) [7]. Ag(QFS)2 rapidly increased in frequency in two replicate populations, reaching complete fixation (100% gene drive carriers in the population) by generations 7 for cage A, and 6 for cage B, leading to a complete suppression in reproductive output by generations 8, and 7, respectively, due to the absence of fertile females (Fig 7C).

## Discussion

Resistance poses an important challenge to the development and potential implementation of gene drives. New methods to mitigate resistance have allowed the successful modification of caged laboratory populations using gene drive systems, however, bridging expectations between lab and field must anticipate a massive increase in population size. Here, we present a novel approach to assess the likelihood of resistance arising in large populations as an essential predictor of gene drive robustness and efficacy. Our strategy considers variants present in natural populations, and those generated through the mutagenic activity of Cas9. The improved throughput of our experimental set-up allowed us to show that one of the most functionally constrained gene drive target sites in the mosquito genome [20,23] can evolve functional alleles, resistant to gene drive activity, where none had been detected before, in cage invasion experiments [7,9].

We find that the only SNP present at an appreciable frequency in natural populations is fully susceptible to Ag(QFS)1 gene drive activity and therefore does not constitute a resistant allele. However, given the limited and non-uniform sampling of sequenced African malaria mosquito populations [22], we cannot exclude yet-to-be-detected resistant alleles residing at significant frequencies in malaria vector species.

By applying a novel genetic screen to enrich for Cas9-induced mutations that encode a functional *dsx* gene product, we recovered ~4,000 mutations that may have arisen independently. This marks a dramatic increase in screening power over single-generation gene drive crosses, or caged population experiments, where typically <1% of the individuals are expected to carry a target site mutation [7,9,17,38–40]. As an example, given the high homing rates for Ag(QFS)1 (93%, S1 Data), and EJ mutations being present in approximately half the non-drive progeny of heterozygote gene drive carriers [7], over ~500,000 gene drive offspring would need to be screened in order to have a power similar to our assay.

Through our mutagenesis screen we discovered, and later validated, the first **R1** allele observed at the Ag(QFS)1 target site that is fully resistant to gene drive activity. Additionally, we found an allele exhibiting incomplete resistance to gene drive, termed **R3**, that is only partially cleavable by Ag(QFS)1. Whilst both alleles maintained full DSX^F^ functionality as assessed through female fertility assays, both alleles harbor nonsynonymous SNPs that may alter DSX function or RNA splicing patterns in subtle ways that we were not able to capture here. A minute fitness cost might explain why these SNPs have not yet been detected in natural populations [22].

When seeded into caged populations, the **R1** allele prevented suppression by Ag(QFS)1, consistent with previous research on resistance [11,17]. In contrast, seeding with the **R3** allele did not prevent gene drive spread, but did prevent complete population suppression.

Indeed, in the presence of **R3**, suppression drives are predicted to gradually level off to establish a stable equilibrium between gene drive and **R3** alleles. This outcome could sustain a moderate level of long-term population suppression (~60%), assuming a very large panmictic population. We speculate that intermediate population suppression would still have epidemiological impact, reducing malaria cases, similarly to insecticide use. However, spatial modeling predicts that even a gene drive facing no resistance is unlikely to spread to fixation [41]. Therefore, **R3** alleles should be viewed with caution, similarly to **R1**. Further modeling efforts considering the evolution of resistance to gene drive and also incorporating: species-specific mosquito population dynamics and spatial structure, malaria disease transmission, and non-gene drive interventions across different geographies in Africa, are needed to determine the level of sustained population suppression required in different contexts, as a minimum, to achieve disease eradication [44].

Importantly, our experimental approach allowed us to calculate the rate of creation of functionally resistant mutations (whether **R1** or **R3**) at 4.1*10^−3^ among all EJ alleles. Considering the rate of homing for Ag(QFS)1, the overall frequency among the offspring of gene drive carriers will be substantially lower at approximately 7.2*10^−5^ (S1 Data). At these rates, the likelihood of resistance to a gene drive like Ag(QFS)1, with only a single target site, exceeds 5% for populations greater than 600 individuals, and thus, it is a near certain outcome in natural populations.

Multiplexed gene drives are expected to reduce the likelihood of resistance by several orders of magnitude, if targeted to several non-overlapping sites showing strong sequence constraints [38,40,42]. To this end, we designed and tested multiplexed derivatives of Ag(QFS)1, named Ag(QFS)2 (two targets) and Ag(QFS)3 (three targets).

The multiplexed design serves to actively remove resistant variants if at least one site remains cleavable. Thus, multiplexing aims to prevent population-level accumulation of single-site variants that can retard gene drive spread, and also the creation of a fully resistant allele through the gradual accumulation of single-site mutations.

For our multiplexed gene drives, Ag(QFS)2 and Ag(QFS)3, we observed high rates of inheritance (>99%) that were at least equivalent to or higher than those of the single-target gene drive Ag(QFS)1. In contrast to previous observations [38,42], we did not see a reduction in homing when one of the target sites was blocked by the **R1** allele, or by an **R2** (GFP) insertion. This is in accordance with recent findings in *An. stephensi*, whereby multiplexing served to override known resistance at the *cardinal* locus, without compromising homing efficiency [43].

The precise mechanistic basis underlying the high transmission rates of our multiplexed drive system—whether through HDR-mediated gene conversion, meiotic drive (elimination of cleaved chromosomes), or a combination of both—remains unresolved and warrants further investigation. Alternative strategies, such as ‘double-tap’ or ‘additive multiplexing’, where gRNAs act sequentially [43], have been proposed, specifically to minimize meiotic drive contributions. By contrast, our strategy targets multiple independent wild-type sites simultaneously, conferring robustness against resistance at any individual locus without requiring prior knowledge of which site may be affected, a property we consider essential for field deployment.

Consistent with previous observations at *dsx* and other gene drive targets [7,17], we observed a fitness cost in Ag(QFS)1-heterozygous females that could be rescued when the gene drive was balanced against the **R1** allele, which could not be cleaved. Α similar rescue of Ag(QFS)1 female fitness was observed in the presence of an anti-CRISPR protein that directly binds and inhibits Cas9 activity [29,44].

Unexpectedly, Ag(QFS)2 females exhibited improved fitness compared to Ag(QFS)1. The two gene drive strains differ in three key aspects: (i) the number of gRNA cassettes present in each gene drive element; (ii) the presence or absence of the coding portion of *dsx* exon 5 between target sites T1 and T3; and (iii) the orientation of the transgene relative to the *dsx* gene locus. By isolating the Ag(QFS)3 strain from five independent founders, we showed that female carriers of gene drive elements showed reduced fitness when the Cas9 and gRNA cassettes were integrated in the same orientation as *dsx*. Differences in fitness might be the result of differential leakiness of Cas9 in somatic tissues, causing the observed mosaic phenotypes [17,29,44], potentially due to directional enhancers [45,46]. Alternatively, differential disruption of *dsx* splicing, and the production of aberrant isoforms that interfere with wild-type protein dimerization might occur, primarily when the constructs are integrated in the same orientation as *dsx*. Dominant negative fitness effects due to partial *dsx* splicing have been hypothesized for gene drive strains in *An. stephensi* [47,48], and reported in *Drosophila suzukii* [49] and *D. melanogaster* [50], where females expressing gene drive constructs displayed intersex and sterile phenotypes.

Our effectively deterministic modeling suggests that an increase in fitness of female gene drive carriers from ~0.2 to ~0.8, is expected to have great impact upon population suppression outcomes in the presence of **R2** alleles, similarly to previous findings [18,19]. However, in the presence of **R1** or **R3** resistance, population suppression outcomes are similar, independent of female fitness: no suppression in the presence of **R1** and a ~60% reduction in vector population size in the presence of **R3**. Interestingly, recent modeling predicts that a fitness cost of up to ~0.6 could be tolerated by a *dsx* gene drive without compromising population suppression [41].

Targeting highly constrained sequences brings the rate of resistance below the threshold of detection in lab-contained releases. Our experimental approach allows such alleles to be created, and quantified, by first selecting for resistance at each site individually, and then combining variants onto a single synthetic, multi-resistant allele for testing. Assuming equivalent rates of resistance at each target site, our modeling determined that gene drives carrying 2 or 3 gRNAs could suppress effective population sizes of up to $\text{3}.\text{6}\times {\text{1}\text{0}}^{\text{7}}$ and $\text{5}.\text{2}\times {\text{1}\text{0}}^{\text{1}\text{1}}$, respectively. Estimates of the effective population size of *An. gambiae* in Africa varies widely, from$\;{\approx \text{1}\text{0}}^{\text{7}}$ based on nucleotide diversity and demographic history inference methods [23], to $\approx \text{2}\times {\text{1}\text{0}}^{\text{8}}\;$based on the analysis of a recent soft sweeps of insecticide resistance alleles [32]. This would indicate that 3 gRNAs would be sufficient to ensure protection against the evolution of resistance.

However, cage experiments conducted in laboratory settings, as in the present study, fail to capture the ecological complexity of natural mosquito populations. In the future, we aim to bridge this gap by testing promising strains under conditions that imitate natural settings, including overlapping generations and temperature fluctuation.

Our models are also simplified to assume a panmictic population [32], without standing variation, whilst ignoring seasonality and complex spatial dynamics. Indeed, spatial models predict that persistent wild-type populations can arise under certain parameter conditions, where demographic stochasticity is enhanced [41,51–53]. Further field surveys are needed to understand the complex interspecific anopheline population structures across different geographies in Africa, the rate of transgenic mosquito dispersal within these populations [54], as well as the broader arthropod biodiversity in malaria endemic regions [55]. Ultimately, we require models that better predict the outcomes of heterogeneous population suppression, informing on the efficacy of a gene drive intervention, and its ecological impact.

## Conclusions

All suppressive technologies, from insecticides to antibiotics, are expected to exert evolutionary pressure for the selection of resistance against them. Since the inception of homing-based gene drive, theoretical exploration has considered different scenarios of resistance emerging, yet experimental evidence has been lacking [10,14,16]. By experimentally accelerating the emergence and selection of resistance, to the best-performing population suppression gene drive to date [7,9], we have been able to make tangible and specific predictions around the scale and rate of resistance emergence at a highly constrained target—the female-specific exon of *dsx*. Such predictions, which are essential in moving from lab to field, were not possible with previous approaches. Finally, we demonstrate that multiplexed gene drives can actively remove resistant alleles, and when targeted to highly constrained loci such as *dsx*, they are predicted to counteract resistance across large populations of the malaria mosquito. Our study should inform the future design of multiplexed gene drives to overcome resistance.

## Materials and methods

### Analysis of standing variation at putative gene drive target sites

The Ag1000 phase 3 data release was accessed from Google Collaboratory, following instructions on the MalariaGEN website (https://www.malariagen.net/data/ag1000g-phase3-snp). The dataset includes genome-wide SNP calls from whole-genome sequencing of 2,784 wild-caught mosquitoes collected from 19 countries [22].

### Mosquito maintenance and microinjections

*An. gambiae* G3 strain wild-type and transgenic mosquitoes were reared at 26 ± 2 °C and 65 ± 10% relative humidity. Larvae were maintained in trays with 500 ml salt water, at a larval density of 200 per tray and fed on NishiKoi food pellets. Adults were fed on glucose and females were blood-fed on cow blood, 5–7 days after being crossed to males, using Hemotek membrane feeders [9]. Microinjections were performed on freshly laid embryos as previously described [7,56]. All plasmids were microinjected at a concentration of 300 ng/μl.

### Generation of an autosomal editor strain

To generate an autosomal editor strain expressing *zpg::Cas9* and U6::dsx-T1 gRNA and targeting the same site on *doublesex* (T1) as the Ag(QFS)1 gene drive we microinjected G3 strain wild-type embryos with the previously described p17410 plasmid [7], together with a vasa-regulated *piggyBac* transposase helper plasmid [57]. The resulting strain is marked by *3xP3::DsRed* and expresses a U6::gRNA complementary to *dsx* T1 along with a *zpg::Cas9* that was insufficient in producing significant levels of *dsx* mutagenesis.

### High-throughput mutagenesis screen at *dsx*

To induce *dsx* T1 mutagenesis, an F1 cross of >100 heterozygous DsRed^+^ males carrying an autosomal editor expressing *zpg::Cas9* and U6::dsx-T1 gRNA, to >100 heterozygous *vasa2::Cas9* YFP^+^ females [4] was performed 5 times, and females of each cross were blood-fed 5, 10 and 15 days after being crossed to produce offspring. 4 days post-bloodmeal (PBM) 4,000–12,000 L1 offspring of the F1 cross were screened and their DsRed^+^YFP^−^ subsection was selected using a complex object particle analyzer and sorter (COPAS) [58]. Fifty DsRed^+^YFP^−^ males were crossed to 50 GFP^+^ null heterozygous females (*dsxF*^*−*^) [7] in an F2 cross. The F2 cross was performed 33 times to balance Cas9-induced mutations across the known null allele in their progeny and examine whether they can restore *dsx* functions in females. Each F2 cross was blood-fed once, and 4 days PBM 2,000–6,000 L1 offspring were screened and their GFP^+^ subsection was selected using COPAS [58]. GFP^+^ mosquitoes were reared to adulthood and 5 days post-emergence they were offered a blood-meal. Subsequently, they were knocked-out using CO_2_ to examine their anatomy and manually sorted into 4 different pools per parental cage: males, intersex, blood-fed (BF) females, non-BF females (S2 Data). Intersex mosquitoes were identified by the presence of semi-plumose antennae and unrotated claspers [7] (S6 Fig). A pool of 100 intersex mosquitoes was analyzed from each parental cage by pooled amplicon sequencing. Mosquitoes that developed normally as females were individually analyzed by Sanger sequencing. Note that samples were individually analyzed several months after being collected due to interruption of the study by the Covid-19 pandemic.

### Pooled amplicon sequencing

Pools of adult mosquitoes (100 intersex mosquitoes per pool for each of the resistance assay cages and ~250 mosquitoes in duplicate per pool for each of the cage trial generations) were subjected to gDNA extraction using the Promega Wizard Genomic DNA Purification kit, PCR amplification under non-saturating conditions as previously [7,17], using primers Illumina-AmpEZ-4050-F1 (ACACTCTTTCCCTACACGACGCTCTTCCGATCTACTTATCGGCATCAGTTGCG) and Illumina-AmpEZ-4050-R1 (GACTGGAGTTCAGACGTGTGCTCTTCCGATCTGTGAATTCCGTCAGCCAGCA), Illumina pooled amplicon sequencing (AmpEZ service, Genewiz, Azenta Life Sciences) and analysis using CRISPResso2 [59]. Datasets were subsequently handled on Python to rename mis-labeled alleles that were falsely grouped together by CRISPResso. A detection threshold for mutations was set at 0.5%.

### Sanger sequencing

Single mosquito samples were subjected to gDNA extraction using the DNeasy Blood & Tissue kit (Qiagen), PCR amplification using primers dsx-intron4-F1 (GTGAATTCCGTCAGCCAGCA) and dsx-exon5-R4 (AACTTATCGGCATCAGTTGCG), and Sanger sequencing by Eurofins Genomics using primer dsx-exon5-R2 (TGAATTCGTTTCACCAAACACAC). Sequence alignments were performed on Benchling.

### Generation and phenotype assessment of the *dsx* variant strains

The three marker-less variant strains containing different SNPs at the *dsx* T1 site (G → A, C → T or G → T) were generated and isolated using CriMCE as previously described [28]. Female fertility of SNP homozygotes and heterozygotes was determined by double-blinded assays, whereby mixed populations of wild-type and SNP carrier females were crossed to wild-type males and allowed to lay eggs individually 2–3 PBM. Eggs and larvae were counted no more than 1 day after they were laid or hatched, respectively. The genotype of each mother was then determined by Sanger sequencing (wild-type, heterozygous or homozygous for each SNP). To determine the extent to which each SNP was cleavable by the gene drive, SNP carrier females were crossed to DsRed^+^ Ag(QFS)1 gene drive males and their *trans-*heterozygote Ag(QFS)1/SNP offspring, as well as Ag(QFS)1/wt individuals of both sexes, were crossed to wild-type. Females were allowed to lay eggs individually 2–3 days PBM. The rate at which Ag(QFS)1 was inherited amongst the offspring was determined through fluorescence microscopy by tracking the DsRed marker. The egg and larval output of Ag(QFS)1/C → T, Ag(QFS)1/G → T and Ag(QFS)1/wt females was also recorded and compared to a simultaneous wild-type control, respectively.

### Population invasion experiments of Ag(QFS)1 with seeded resistance

Population invasion experiments were set up by seeding wild-type populations with either **R1** or **R3**, and the Ag(QFS)1 gene drive. Briefly, **R1**-seeded populations were setup in duplicate cages, initiated with 240 Ag(QFS)1 heterozygous gene drive males (40% genotype frequency) and a mixed population of 306 wild-type and 54 **R1** homozygotes (9% allelic frequency). **R3**-seeded populations were setup in duplicate cages, initiated with 150 Ag(QFS)1 heterozygous gene drive males (25% genotype frequency) and a mixed population of 450 mosquitoes carrying **R3** at approximately 25% allele frequency (the progeny of **R3** heterozygous females crossed to wild type).

The populations were mixed as pupae in the starting generation (G0) and were allowed to emerge in the same cage. Adult mosquitoes were left to mate for 5 days post-emergence, before being blood-fed on cow blood using a Hemotek membrane feeding system for 30 min. Filter paper-lined cups of water were placed in the cages 2 days PBM. Eggs were collected and photographed 3 days PBM. 1st instar larvae (L1) were screened 4–5 days PBM, by tracking the DsRed fluorescence associated with the gene drive through fluorescence microscopy or by using the COPAS larval sorter [58]. A minimum of 600 larvae were randomly selected and split into 3 trays of 500 ml water (200 larvae per tray). All surviving pupae were collected and transferred to a new cage for the adults to emerge and establish the following generation. A representative portion of these (100–300 individuals) were screened as pupae to determine the sex ratio per generation (S3 Data). Note that males and intersex are indistinguishable at the pupal stage, however, mosaic intersex females and phenotypically typical females can be distinguished (S3 Data). After blood-feeding and oviposition of each following generation the adults of the previous generation were collected and stored in the −20 °C for further analysis by pooled amplicon sequencing to determine the frequency of **R1** and **R3** alleles in each cage.

### Modeling

We perform two types of simulations: (i) effectively deterministic simulations that include a partially resistant allele **R3** with Beverton-Holt population dynamics (Fig 4) and (ii) stochastic simulations of cages that are maintained at a fixed (Fig 3B) or varying population size (Fig 5). Both use the same underlying stochastic modeling framework, as previously described [16], and are based on a Wright-Fisher model for population genetics with two separate sexes. For simulations of type (i), we use a very large initial population size ($N_{e}={\text{1}\text{0}}^{\text{1}\text{2}}$) that mimics deterministic dynamics, which use a very accurate and computationally efficient approximation to multinomial sampling [16]; however, as a result of the population dynamics, when the total population size is small this results in stochastic effects just before population elimination, as such in the instances where the population size got drastically reduced and the populations eliminated, we have only plotted allele frequencies for generations in which $N_{e}>\text{1}\text{0},\text{0}\text{0}\text{0}$. For simulations of type (ii), we use a fixed small population size of $N_{e}=\text{6}\text{0}\text{0}$ (Fig 3B) or varying population sizes from $N_{e}=\;$1^00^ up to $N_{e}=$ 10^16^ to determine the critical population size ($N_{max}$) that can be eliminated by a gene drive with a single, two or three target sites with 95% confidence (Fig 5). For simplicity, a standard fitness cost of 0.76 (i.e., the fitness cost of Ag(QFS)1 heterozygous females) was applied for any female gene drive carrier, since fitness only impacted upon the critical population size calculation for a single-target gene drive, but not for a gene drive containing two or three target sites. To account for the observed initial advantage of Ag(QFS)1 males in the experimental **R3** cages, due to the separate rearing of Ag(QFS)1 and wt/R3 individuals, we started the initial Ag(QFS)1 frequency at 0.6 in the simulations to approximately match the cage data (Fig 3B).

The key difference between the simulations in this paper and previous modeling of resistance to gene drive [16], is the inclusion of the partially resistant allele **R3**. The process of gametogenesis modeled in the simulations is shown in S9 Fig, with parameters as described in S2 Table and S18 Fig. Note that the only genotypes are inherited in a super-mendelian fashion are W\D (Ag(QFS)1/wt) and R3\D (Ag(QFS)1/R3). We assume that **R1** and **R2** alleles completely block gene drive homing.

### Estimating $N_{max}$ from probability of resistance curves

Given data from simulations of the probability of resistance $p_{R}$ at different gridded values of population size N, we numerically estimate the value $N_{max}$—the value at which the probability of resistance crosses 0.05, using the MATLAB functions griddidInterpolant to give a interpolant object of the shifted data $p_{R}-\text{0}.\text{0}\text{5}$ and then fzero to find the zero (MATLAB version: 24.1.0.2603908 (R2024a), Natick, Massachusetts: The MathWorks; 2024). For better fidelity this is performed for log-scaled values of *N*.

### Cloning of CRISPR constructs with multiplexed gRNAs

Primers containing *Bsa*I sites (capitals) and gRNA sequences (capitals): BsaI-T1-U6-F (gagGGTCTCatgctGTTTAACACAGGTCAAGCGGgttttagagctagaaatagcaagt) and BsaI-T3-U6-R (gagGGTCTCaaaacCTCTGACGGGTGGTATTGCagcagagagcaactccatttcat), were used to amplify a gRNAscaffold-U6terminator-U6promoter cassette from plasmid p131 [28]. The resulting PCR product was inserted into the p174 master-vector previously described [7], through GoldenGate cloning to create vector p174102 that was used as a helper plasmid to generate the Ag(Dsx) docking line, and as a donor vector to generate the Ag(QFS)2 gene drive. This vector (p174102) contains a *zpg::hCas9* nuclease cassette, *3xP3::DsRed::Sv40* marker, and two tandemly repeated *U6::gRNA* cassettes targeting sites T1 and T3 on *dsx* exon 5 (S7 Fig). To create the p174103 vector for generation of the Ag(QFS)3 strain, oligonucleotide sequences: dsx-T2-F (TGCTGTCTGAACATGCTTTGATGGCG) and dsx-T2-R (AAACCGCCATCAAACATGTTCAGAC) were annealed and cloned into the p139 vector containing a gRNAscaffold-U6terminator-U6promoter-BsaIcloningsite-gRNAscaffold-U6terminator-U6promoter cassette, through GoldenGate cloning. The resulting plasmid was amplified using primers BsaI-T1-U6-F and BsaI-T3-U6-R like before, to enable GoldenGate cloning of the PCR product into p174, to create p174103, containing a *zpg::hCas9* nuclease cassette, *3xP3::DsRed::Sv40* marker, and three tandemly repeated *U6::gRNA* cassettes targeting sites T1, T2 and T3 on *dsx* exon 5 (S7 Fig).

### Cloning of donor plasmids to generate the Ag(Dsx) gene drive docking line

The pDLv3 donor vector, used for generation of the Ag(Dsx) strain, was created by Gibson assembly. A 3xP3::GFP::SV40 marker cassette flanked by attP sites was amplified using primers 4050-KI-Gib34 (ATGTTTAACACAGGTCAAGACCGGTACCCCAATCGTTCA) and 4050-KI-Gib35 (GCGGAAAGTTTATCATCCACTCACGCGTTCAGGATTATATCT). Genomic regions 1.8 kb upstream and downstream of the dsx intron4-exon5 splice junction were amplified using primer pairs 4050-KI-Gib1 (GCTCGAATTAACCATTGTGGACCGGTCTTGTGTTTAGCAGGCAGGGGA) with 4050-KI-Gib33 (TGAACGATTGGGGTACCGGTCTTGACCTGTGTTAAACATAAATG), and 4050-KI-Gib36 (AGATATAATCCTGAACGCGTGAGTGGATGATAAACTTTCCGCAC) with 4050-KI-Gib4 (TCCACCTCACCCATGGGACCCACGCGTGGTGCGGGTCACCGAGATGTTC), to make up the left and right homology arms of the donor plasmid, respectively. The pK101 plasmid [7] was digested with restriction enzymes *Mlu*I and *Bsh*TI to release its 5.4 kb backbone containing a 3xP3::DsRed::SV40 marker, which was combined with the three amplicons described, in a 4-fragment Gibson assembly reaction, so that the *dsx* homology arms get placed to each side of the attP*-*flanked eGFP cassette.

### Generation of the Ag(Dsx) docking strain

To generate the Ag(Dsx) docking line to accommodate recombinase-mediated cassette exchange (RMCE) of plasmids p174102 and p174103 to generate the Ag(QFS)2 and Ag(QFS)3 gene drive constructs, respectively, wild-type embryos of the *An. coluzzii* G3 strain were microinjected with the p174102 CRISPR plasmid and the pDLv3 donor plasmid. In transformants, CRISPR elements expressed by p17402 caused excision of the region between the T1 and T3 cut sites on *dsx* exon 5, and its replacement with the attP*-*flanked GFP marker cassette from the pDLv3 plasmid, facilitated by HDR. All microinjection survivors (G0) were outcrossed to wild-type mosquitoes and positive transformants were identified through fluorescence microscopy as GFP^+^ and DsRed^−^.

### Generation of the Ag(QFS)2 and Ag(QFS)3 multiplexed gene drive strains

To generate the Ag(QFS)2 and Ag(QFS)3 multiplexed gene drive strains, heterozygotes of the Ag(Dsx) strain were crossed to each other, and their progeny were microinjected with the p174102 or p174103 CRISPR plasmids, respectively, together with a vasa-integrase helper plasmid [57], to facilitate RMCE of the GFP cassette of Ag(Dsx) for each of the gene drive constructs. All microinjection survivors (G0) were outcrossed to wild-type mosquitoes and positive transformants were identified through fluorescence microscopy as DsRed^+^ and GFP^−^. Construct orientation was determined via PCR using primer pairs zpg-term-F1 (GCTGTACTACATCTCGTGGACG) with dsx-exon5-R2, which would only produce an amplicon if the gene drive construct is integrated the same orientation as the *dsx* gene (fwd), and zpg-term-F1 with dsx-intron4-F1, which would only produce an amplicon if the gene drive construct is integrated in reverse (rev) with respect to *dsx*.

### Phenotype assessment of multiplexed gene drive strains

Gene drive homing of strains Ag(QFS)2 and Ag(QFS)3 was assessed by crossing heterozygous DsRed^+^ gene drive parents of both sexes that inherited the drive from either males or females to wild-type. Ag(QFS)2 heterozygous individuals (DsRed^+^) of both sexes, were additionally crossed to heterozygous individuals of a GFP-marked *dsx*F^−^ strain [7], where a GFP cassette interrupts target site T1 but leaves T3 exposed to cleavage. Trans-heterozygous GFP^+^ DsRed^+^ males (since females were sterile due to carrying a homozygous *dsxF*^*−*^ disruption) were crossed to wild-type females to determine rates of gene drive inheritance in the offspring. In a similar fashion, Ag(QFS)2 heterozygous males were crossed to females homozygous for the T1-4G→T SNP which constitutes an **R1** allele, resistant to gene drive cleavage at target site T1. Ag(QFS)2/R1 *trans-*heterozygotes of both sexes were then crossed to wild-type to determine the rate of gene drive inheritance in their offspring via cleavage of the T3 target site alone.

After allowing females to lay eggs individually, 2–3 days post-bloodmeal, the rate at which each gene drive (DsRed^+^) was inherited amongst their progeny was determined through fluorescence microscopy. The fertility of Ag(QFS)1, Ag(QFS)2 and wild-type females was compared through fertility assays performed simultaneously to allow direct comparison. These looked at egg and larval output, mating ability and post-bloodmeal mortality. Females that inherited each drive from either males or females, as well as wild-type females, were crossed to wild-type males, and allowed to lay eggs individually 2–3 days post-bloodmeal. Their egg and larval output were counted no longer than 1 day post laying and hatching, respectively. The number of females that had died in any of the 3–4 days post-bloodmeal were recorded. Females were also interrogated for their mating ability by examining their spermathecae for sperm presence under an EVOS high-resolution light microscope. Note that previously, phenotypic assays of Ag(QFS)1 excluded from the analysis females that were unmated and females that did not blood-feed [7]. In the present study all females were included in the analysis to estimate a more accurate measurement of female fertility, since blood-feeding and mating ability might be affected by the *dsx*F knockout. Finally, the number of females showing a mosaic intersex phenoype amongst progeny of males of different gene drive strains that were integrated in forward (fwd) or reverse (rev) orientation with respect to the dsx gene, were counted. This included Ag(QFS)1 (fwd orientation), Ag(QFS)2 (rev orientation) and five independently created Ag(QFS)3 strains of either orientation.

### Cohort survival of Ag(QFS)2 heterozygotes

Triplicate trays were set up, each containing 50 Ag(QFS)2 heterozygous L1 larvae mixed with 50 wild-type L1 larvae (100 individuals in total per tray). Ag(QFS)2 and wild-type larvae were therefore reared together in each triplicate experiencing the same conditions, whilst also competing for resources. Individuals were screened after they pupated (5–6 days later), and the numbers of live Ag(QFS)2 (DsRed^+^) and wild-type (DsRed^−^) pupae were recorded. Pupae from each tray were added into the same cage, but in separate cups based on their fluorescence. Once all pupae had eclosed (2 days later), the cups were removed from the triplicate cages and the number of individuals that died as adults or failed to eclose and died as a result were recorded.

### Mating competitiveness of Ag(QFS)2 males

Triplicate cages were set up, each containing 50 Ag(QFS)2 (DsRed^+^) heterozygous male pupae, 50 wild-type (DsRed^−^) male pupae and 60 wild-type female pupae, so that males (being 100 in total per triplicate) compete for mating with the females. All pupae eclosed within 24 hours, and were allowed to mate a further 4 days, after which point adult females were blood-fed. 2 days later 50 females from each cage (150 females in total from all triplicates) were placed in individual cups to lay eggs, and 4 days later the identity of their offspring was recorded (DsRed^+/−^) to figure out whether they had mated with a Ag(QFS)2 heterozygote wild-type male.

### Population invasion experiments of Ag(QFS)2 in small cages

Population invasion experiments were performed in duplicate with each G0 population consisting of 300 wild-type females, 150 wild-type males and 150 Ag(QFS)2 DsRed^+^ heterozygous males that inherited the gene drive paternally, as previously [7]. The initial caged release was performed using age-matched pupae. Mosquitoes were left to mate for 5 days post-emergence before being blood-fed on cow blood. Two days later they were allowed to lay eggs. 650 eggs were randomly selected to seed the next generation,

whilst the rest were photographed and counted using JMicroVision. Emerged larvae were screened for the presence of DsRed, indicative of the gene drive, using fluorescence microscopy (S4 Data). After pupation the percentage of phenotypic females amongst all pupae including males/intersex and mosaic intersex, was recorded to give an indication of the reproductive capacity of the caged populations, before allowing all pupae indiscriminately to seed the next generation (S4 Data). Note that sterile intersex mosquitoes are indistinguishable from males at the pupal stage.

### Statistical analyses

All statistical analyses were performed using Graphpad Prism 10, including normality tests and determination of statistical significance, by calculating *p*-values and effect sizes with 95% confidence intervals (95% CIs). For Kruskall–Wallis statistical comparisons with Dunn’s post-hoc test for multiple comparisons the 95% CIs were calculated manually by first determining the standard error (SE) of each comparison using the formula: $SE=\;\sqrt{\frac{N(N+\text{1})}{\text{1}\text{2}}(\frac{\text{1}}{n_{\text{1}}}+\frac{\text{1}}{n_{\text{2}}})}$, where *N* is the total number of observation across both samples, *n*_1_ is the size of the first sample and *n*_2_ is the size of the second sample. The term $\frac{N(N+\text{1})}{\text{1}\text{2}}$ represents the expected variance of the ranks under the null hypothesis (no difference between the groups). Then the upper and lower 95% Cis were calculated using the formula: 95% CI=mean rank difference±1.96*SE, where 1.96 is the z-score corresponding to a 95% confidence level, which assumes the sampling distribution is approximatelynormal.

### Data visualization

Graphs were plotted on Graphpad Prism 9 and Prism 10, and illustrations were designed using Biorender (full licence), Sankeymatic (https://sankeymatic.com/), Natural Earth (https://www.naturalearthdata.com) and Adobe Illustrator.

## Supporting information

S1 Fig The generation of drive-resistant mutations. **(A)** There are four main repair outcomes after a gene drive-derived Cas9 and gRNA catalyze cleavage of an exposed chromosome, carrying their intact target site, in the germline. The most common outcome is homing of the gene drive (light brown) due to homology-directed repair (HDR). Alternatively, the cut chromosome gets repaired by end-joining (EJ). Perfect EJ repairs the wild-type allele (light gray), which is susceptible to further cleavage. EJ is often error-prone and can lead to the introduction of cut-resistant mutations at the gene drive target site that are either functional (R1, red) or non-functional (R2, blue). Functional resistance (R1) has a selective advantage and can reverse gene drive spread. **(B)** The *doublesex* gene is expressed into two sex-specific isoforms. Targeting the highly conserved, and presumably functionally constrained region on the intron-exon boundary of the female-specific exon (exon 5) of *dsx*, can limit R1 alleles. **(C)** Indeed, no R1 alleles were detected in caged laboratory populations, leading to complete population elimination in less than a year [7,9]. However, natural populations are larger, by several orders of magnitude, which increases the likelihood of drive-induced R1 allele formation, whilst R1 alleles might also be pre-existing in nature. The figure was created in BioRender. Morianou, I. (2026) https://BioRender.com/12gfa3p.
(TIF)

S2 Fig Multiplexed gene drives can mitigate resistance by targeting multiple sites simultaneously. **(A)** Resistant mutations (shown in red) are removed from the target locus provided that one of the targeted sites remains cleavable, to permit homing. **(B)** To block gene drive homing all target sites would need to carry a resistant mutation (shown in red). The figure was created in BioRender. Morianou, I. (2026) https://BioRender.com/81igf6f.
(TIF)

S3 Fig Natural variation on the coding sequence of the female-specific exon of *doublesex.* **(A)** Multiple *Anopheles* species alignment reveals a high amount of nucleotide conservation in both the coding region of exon 5 and flanking non-coding regions (intron 4, exon 5 UTR). In bold are species that belong to the *An. gambiae* species complex. Nucleotide changes are highlighted in gray and those leading to amino acid changes are in red, whilst silent changes are in blue. Three sites that could be targeted by gene drive (T1, T2 and T3) are shaded and underlined on the *An. gambiae* reference. Variable nucleotides within them, as identified by analyzing the phase 3 Ag1000G project data are highlighted in yellow. Their proto-spacer adjacent NGG motifs (PAM) are underlined in red. The dashed line pinpoints the cleavage sites. **(B)** The table shows single nucleotide polymorphisms (SNPs) that were identified amongst 2,784 wild-caught mosquitoes in *An. gambiae, coluzzii* or *arabiensis*, and their relative positions compared to each cleavage site in T1, T2 or T3. Maximum allelic frequency refers to the frequency of each SNP in the country in which it was most frequent. The percentage of mosquitoes that possessed each SNP in heterozygosis (HET) vs. homozygosis (HOM) is also shown.
(TIF)

S4 Fig The allelic distribution of the natural G→A variant (2R:48714641), across Ag1000G sampling locations. The G→A natural variant was found at 1.3% allelic frequency across all sampling locations, including 19 African countries and territories (*N* = 5568 alleles). Specifically, it was found at 1.0% allelic frequency in Cameroon (*N* = 888 alleles), 5.8% allelic frequency in Gabon (*N* = 138 alleles), 7.2% allelic frequency in the DRC (*N* = 152 alleles) and at 25.9% allelic frequency in Angola (*N* = 162 alleles). The SNP’s allelic distribution per country is shown to the top right (*N* = 70 alleles), its species distribution to the middle right (*N* = 64 alleles), and its genotype distribution in Angola (*N* = 81 genotypes), where it was most frequent to the bottom right. The map of Africa was designed using Natural Earth (https://www.naturalearthdata.com/).
(TIF)

S5 Fig By exploiting maternal deposition of Cas9 we can generate high rates of end-joining mutations at the Ag(QFS)1 target site. **(A)** A minimum of 50 U6::gRNA(*Dsx*-T1) DsRed^+^ males were crossed to a minimum of 50 *vas2::Cas9* YFP^+^ females. **(B)** The female offspring of the cross were examined for intersex mosaicism at the pupal and adult stage, visible by the development of male-like physical characteristics, depending on their genotype. The ± signs indicate the presence (+) or absence (−) of the transgenes associated with each given fluorescent marker (DsRed/YFP). The data underlying this figure can be found in S1 Data. Panel A was created in BioRender. Morianou, I. (2026) https://BioRender.com/rvgxqe9.
(TIF)

S6 Fig The intersex phenotype. **(A)** Intersex females develop semi-plumose antennae (blue arrows), resembling that of males; male-like palps, and a proboscis that is unable to draw blood (yellow arrows); and under-developed claspers, which females completely lack, facing upwards, instead of downwards like in males (red arrows) [7]. **(B)** Claspers rotate to face downwards in mature males, whereas intersex claspers remain facing upright. **(C)** Pupal genitalia are denoted using red circles. Fully intersex females develop male-like genitalia (uniform phenotype). Mosaic intersex individuals can be distinguished by their under-developed male-like genitalia (variable phenotype, showing different degrees of penetrance depending on the level of mosaicism).
(TIF)

S7 Fig Distinct heritable mutations that occur at the Ag(QFS)1 target site through embryonic deposition of Cas9 (A) Schematic of F2 single crosses that were set up to answer how many distinct mutations each mutated male parent contributed to its offspring. 8 Mosaic males carrying mutations at *dsx*-T1, due to *vas*-Cas9 maternal deposition, were crossed to females carrying a GFP*-*tagged *dsxF*^*−*^ null allele, in single crosses. **(B)** The portion of distinct mutations inherited amongst their offspring, as determined by Sanger sequencing is shown (each cross is denoted with a distinct sample ID). Eight to ten individuals were examined per clutch. In total,12 distinct mutant alleles were discovered in the 76 individual offspring that were sequenced collectively from all single crosses. The data underlying this figure can be found in S1 Data. Panel A was created in BioRender. Morianou, I. (2026) https://BioRender.com/jo9r5v0.
(TIF)

S8 Fig Sequence alignment of the alleles discovered through the mutagenesis screen at the Ag(QFS)1 target site. Deletions are denoted by dashes, and SNPs are depicted in red (note 75*C→T, designated as **R3**; 74*G→T, designated as **R1**. The T1 gRNA spacer binding site is highlighted in blue and the PAM in gray. The frequency of each depicted allele is shown to the right. **(A)** Alleles discovered in anatomically intersex (sterile) GFP^+^ females. **(B)** Alleles discovered in GFP^+^ females that developed normally, as typical females. The amino acids encoded by codons carrying SNPs are also shown.
(TIF)

S9 Fig The types of Cas9-induced EJ mutations at the Ag(QFS)1 target site, recovered in the intersex fraction of F3 offspring from from distinct F2 crosses of the mutagenesis screen. **(A)** A minimum of 50 mosaic males containing a multitude of Cas9-induced EJ mutations were crossed to a minimum of 50 *dsx* null (*dsxF*^*−*^)-carrying females (GFP^+^) [7]. This cross was performed in replicate cages 33 times (cages A, B, C, D, E, F, G, H, I, J, K, L, M, N, O, P, Q, R, T, U, V, W, X, Z, AC, AD, A20, B20, C20, D20, E20, F20, G20). For each cross, 100 GFP^+^ intersex offspring were analyzed through pooled amplicon Illumina sequencing. The bars show the relative portion of each mutation recovered in intersex individuals (A). The graph shows the allelic frequency of the most common mutation present in each intersex offspring pool **(B)**. As controls, three pools of 100 non-sex-separated individuals were subjected to pooled amplicon Illumina sequencing. The first two pools contained the GFP^+^ offspring of a cross of 50 *dsx* mutator males (expressing both a *zpg-*Cas9 and a gRNA against the target site of Ag(QFS)1) to 50 null GFP^+^ females, in the absence of the maternal *vas2::Cas9* strain. Offspring that had also inherited the *dsx* mutator allele (mutator^+^, ALEDL), in addition to the *dsxF*^*−*^ GFP^+^ null allele, were analyzed separately from those that did not inherit the *dsx* mutator allele (mutator^−^, DL). The third pool only contained wild-type individuals (WT). The data underlying this figure can be found in S2 Data and https://doi.org/10.5281/zenodo.20541932.
(TIF)

S10 Fig Amplicon sequencing of anatomical females carrying putative drive-resistant mutations. These samples were previously shown to carry a single allele paired to the null *dsx* mutation through Sanger sequencing: the C→T SNP, designated as **R3** (majority light orange), the G→T SNP, designated as **R1** (majority red) or a WT allele (majority dark gray). F3 GFP^+^ individuals were batch-collected from large cages containing >500 mosquitoes and separated on a CO_2_ pad into three groups comprising of: (1) males, (2) anatomical females, and (3) anatomical intersex, for long-term storage (>6–12 months). Prior to gDNA extraction anatomical females were individually separated. Note that low level of cross-contamination of the samples is possible. The data underlying this figure can be found in S1 Data and https://doi.org/10.5281/zenodo.20545056.
(TIF)

S11 Fig Fertility of females carrying each of the SNP variants engineered at the target site of Ag(QFS)1. **(A)** Egg and larval output of females carrying the naturally occurring G→A SNP (non-resistant). The data are not normally distributed (D’Agostino–Pearson normality test). Mann–Whitney non-parametric test: ns: not significant (with *p-*value = 0.3051, HL diff. = 7.00, 95% CI: *−*6.00 to 31.00 for Hom vs. WT egg output; and *p-*value = 0.6459, HL diff. = 0.00, 95% CI: *−*14.00 to 29.00 for Hom vs. WT larval output). **(B)** Egg hatching rate of the offspring of females carrying the naturally occurring G→A SNP. The data are not normally distributed (D’Agostino–Pearson normality test). Mann–Whitney non-parametric test: *: significant with *p-*value = 0.0463, HL diff. = *−*0.04, 95% CI: *−*0.09 to 0.00. **(C)** Egg and larval output of females carrying the Cas9-induced C→T SNP (partially resistant, R3). Only egg output data are normally distributed (D’Agostino–Pearson normality test) and were therefore analyzed using an ordinary ANOVA: ns: not significant, with *p-*value = 0.7869, mean diff. = −5.41, 95% CI: −25.97 to 15.15 for Het vs. WT egg output, and *p-*value = 0.9946, mean diff. = −1.17, 95% CI: −31.12 to 28.77 for Hom vs. WT egg output. Larval output data were analyzed using the Kruskall–Wallis non-parametric test and Dunn’s post-hoc test for multiple comparisons: ns: not significant with *p-*value = 0.4326, 95% CI: −25.37 to 8.50 for Het vs. WT larval output, and *p-*value > 0.9999, 95% CI: −22.49 to 23.78 for Hom vs. WT larval output. **(D)** Egg hatching rate of the offspring of females carrying the Cas9-induced C→T SNP. The data are not normally distributed (D’Agostino–Pearson normality test) and were analyzed using the Kruskall–Wallis non-parametric test, and Dunn’s post-hoc test for multiple comparisons: *: significant with *p-*value = 0.0445, 95% CI: −32.26 to 2.94; ns: not significant with *p-*value > 0.9999, 95% CI: −20.82 to 30.33. **(E)** Fertility of females carrying the Cas9-induced G→T SNP (fully resistant, R1). The data are not normally distributed (D’Agostino–Pearson normality test) and were therefore analyzed using the Kruskall–Wallis non-parametric test, and Dunn’s multiple comparisons post-hoc test: ns: not significant with *p-*value > 0.9999, 95% CI: −21.63 to 18.25 for Het vs. WT egg output; *p-*value > 0.9999, 95% CI: −38.11 to 10.51 for Hom vs. WT egg output; *p-*value = 0.4874, 95% CI: −42.76 to 2.80 for Het vs. WT larval output; and *p-*value > 0.9999, 95% CI: −31.87 to 16.76 for Hom vs. WT larval output. **(F)** Egg hatching rate of the offspring of females carrying the Cas9-induced G→T SNP. The data are not normally distributed (D’Agostino–Pearson normality test) and were therefore analyzed using the Kruskall–Wallis non-parametric test, and Dunn’s multiple comparisons post-hoc test: ns: not significant with *p-*value = 0.4863, 95% CI: −30.23 to 17.01 for Het vs. WT hatching rate; and *p-*value = 0.6497, 95% CI: −18.57 to 30.05 for Hom vs. WT hatching rate. Abbreviations: WT = wild-type (wt/wt genotype), Het = heterozygous for each variant (wt/SNP genotype), Hom = homozygous for each variant (SNP/SNP genotype). The data underlying this Figure can be found in S7 Data, and the corresponding statistical analyses in S5 Data.
(TIF)

S12 Fig Schematics of the gene drive constructs targeting the *dsx* gene, tested in the present study. **(A)** The Ag(QFS)1 gene drive construct integrated in the same orientation as the *doublesex* gene. **(B)** The Ag(QFS)2 gene drive construct integrated in the reverse orientation with respect to *dsx*. **(C)** The Ag(QFS)3 gene drive construct integrated in the reverse orientation with respect to *dsx*. Gene drive components: att: = RMCE ruminant attachment sites, SV40 = viral terminator, RFP = DsRed fluorescent marker, 3xP3 = neuronal promoter, zpg = *zero population growth* promoter and untranslated region (UTR), Cas9 = human codon-optimized *Streptococcus pyogenes* Cas9 (SpCas9) gene, U6 = pol III promoter and terminator, T1/2/3 = gRNAs containing spacer sequences complementary to sites T1, T2 or T3. The figure was created in BioRender. Morianou, I. (2026) https://BioRender.com/p42nzim.
(TIF)

S13 Fig Fitness of Ag(QFS)2 (A–C) and Ag(QFS)3 (D–F) multiplexed gene drive carriers compared to wild-type controls. Male (M) and female (F) heterozygous gene drive carriers that inherited the paternally (M→M, M→F) or maternally (F→M, F→F) were crossed to wild-type, and the number of eggs (A, D) and larvae (B, E) produced per female parent were scored. The hatching rate of the eggs is also shown (C, F). Only mated females are included in the Ag(QFS)2 analysis (A–C) and both mated and unmated females are included in the Ag(QFS)3 analysis (D–F). Mean values, the standard error around the mean (S.E.M.), together with each sample size (n) are shown to the right of each graph, for each cross. The fertility of Ag(QFS)1 and Ag(QFS)3 gene drive carriers was compared to the wild-type control using a Kruskall–Wallis non-parametric test with Dunn’s post-hoc multiple comparisons test, since the data were not normally distributed (D’Agostino–Pearson). The data underlying this Figure can be found in S8 Data, and the corresponding statistical analyses in S5 Data.
(TIF)

S14 Fig Cohort survival of Ag(QFS)2 heterozygotes vs. wild-type mosquitoes. Starting from 50 Ag(QFS)2 and 50 wild-type L1 larvae per tray per triplicate, the number of individuals that survived into becoming pupae and adults were recorded. On average, 85.3% of Ag(QFS)2 L1 larvae pupated, compared to 96.7% of wild-type L1 larvae; and 75.3% of Ag(QFS)2 L1 larvae reached adulthood, compared to 70.7% of wild-type L1 larvae. The data passed the Shapiro–Wilk test for normality and were analyzed using a mixed effects two-way ANOVA, taking into account the paired nature of the data, comparing the Ag(QFS)2 (green) and wild-type (gray) groups across developmental stages (L1→pupae→adults), to find no significant difference between them (*F*(1.476, 5.905) = 1.653, *p-*value = 0.2611). The data underlying this Figure can be found in S8 Data, and the corresponding statistical analyses in S5 Data.
(TIF)

S15 Fig Mating competitiveness of Ag(QFS)2 heterozygotes vs. wild-type males. Fifty Ag(QFS)2 heterozygous males competed against 50 wild-type males for mating with 60 wild-type females, in triplicate. The identity (Ag(QFS)2 in green or wild-type in gray) of the offspring of 50 randomly selected females per triplicate revealed whether those females had mated with Ag(QFS)2 carriers or wild-type males. On average (mean ± SD), a greater fraction of females had mated with wild-type (0.50 ± 0.02) than Ag(QFS)2 males (0.41 ± 0.03), however this difference was not significant as deduced by paired *t* test (*p*-value = 0.0604; 95% CI: −0.010 to 0.197, *η*_p_^2^ = 0.8829), after the data passed the Shapiro–Wilk test for normality. For clarity the fraction of females that did not produce successful matings (0.09 ± 0.03) were also plotted. Means are shown above each column and error bars represent the standard deviation (SD). The data underlying this Figure can be found in S8 Data, and the corresponding statistical analyses in S5 Data.
(TIF)

S16 Fig Fitness of Ag(QFS)2 gene drive carriers, compared to Ag(QFS)1. Female (F) heterozygous individuals carrying either Ag(QFS)1 or Ag(QFS)2, that had inherited each gene drive paternally (M→F) or maternally (F→F) were crossed to wild-type (WT), and the number of eggs **(A)** and larvae **(B)** produced per female parent were recorded. At the same time the egg a larval output of a wild-type cross were recorded to function as a control. The hatching rate of eggs is also shown **(C)**. Note that both mated and unmated females are included in this analysis. Mean values, the standard error around the mean (S.E.M.), together with each sample size (*n*) are shown to the right of each graph, for each cross. The fertility of Ag(QFS)1 and Ag(QFS)2 gene drive carriers was compared to the wild-type control and also to one another using a Kruskall–Wallis non-parametric test with Dunn’s post-hoc multiple comparisons test, since the data were not normally distributed (D’Agostino–Pearson). The data underlying this Figure can be found in S8 Data, and the corresponding statistical analyses in S5 Data.
(TIF)

S17 Fig Intersex mosaicism in female QFS gene drive carriers. **(A)** Example of the female genitalia at the pupal stage, of phenotypic females (top) versus mosaic intersex females (bottom). **(B)** Percentage of intersex mosaics in the female offspring of male gene drive carriers of Ag(QFS)1, Ag(QFS)2 or Ag(QFS)3 that harbored the gene drive in the same (fwd) or reverse (rev) orientation in the genome, with respect to *dsx.* Means and standard deviations are shown above the graph and indicated by the error bars. A *t* test with Welch’s correction to account for unequal standard deviation in the two datasets was performed with *p-*value = 0.0104, *η*^2^ = 0.9671, 95% CI: *−*99.34 to *−*35.20. Note that each data point originated from an independently generated strain (i.e., isolated from a different founder). **(C)** Illustrations of the gene drive integration mode with respect to *dsx* (fwd or rev) for each of the gene drives examined. The data underlying Panel B can be found in S8 Data, and the corresponding statistical analyses in S5 Data. Panel C was created in BioRender. Morianou, I. (2026) https://BioRender.com/q7ssenl.
(TIF)

S18 Fig Diagram of resistant allele creation during gametogenesis, as incorporated into our modeling framework. Abbreviations: EJ = end-joining, D = drive allele,W = wild-type allele, N = non-functional EJ mutation, R = functional resistant mutation, R1 = functional resistant mutation, R2 = non-functional resistant mutation, R3 = functional partially resistant mutation. Model parameters: ε = cleavage efficiency, ε_W_ = cleavage efficiency of wild-type, ε_R3_ = cleavage efficiency of R3, ν = fraction of mutated alleles, β = fraction that are R1, γ = fraction that are R3, β′ = fraction that are R1, γ′ = fraction that are wild-type. The figure was created in BioRender. Morianou, I. (2026) https://BioRender.com/ihvcj9e.
(TIF)

S1 Table The number of genetic females assessed as part of the mutagenesis screen, and the portion that carried putative resistant mutations. (DOCX)

S2 Table Modeling parameters. See also S18 Fig for an illustration of how the modeling parameters relate to each other.
(DOCX)

S1 Data Resistance Screen: Summary Data and Modeling Parameters. (XLSX)

S2 Data Resistance Screen: Sample Composition and Mutant Allele Frequencies. (XLSX)

S3 Data Cage Trials of Ag(QFS)1 with Pre-seeded Resistance. (XLSX)

S4 Data Cage Trials of Ag(QFS)2. (XLSX)

S5 Data Statistical Analyses. (XLSX)

S6 Data Deterministic Modeling: Simulation Outputs. (XLSX)

S7 Data Phenotype assessment of engineered SNP carriers. (XLSX)

S8 Data Phenotype assessment of multiplexed gene drive carriers. (XLSX)

S9 Data Probability of resistance evolving simulations. (XLSX)

## Abbreviations

BF: blood-fed
CriMCE: CRISPR-mediated cassette exchange
EJ: end-joining
HDR: homology-directed repair
PBM: post-bloodmeal
WT: wild-type
