# Supplementary figures and images for: Engineering resilient gene drives for sustainable malaria control by predicting, testing and overcoming target site resistance in Anopheles gambiae
Source: PLoS Biol. 2026 Jul 6;24(7):e3003879. doi: 10.1371/journal.pbio.3003879 (PMC13395382; doi:10.1371/journal.pbio.3003879)

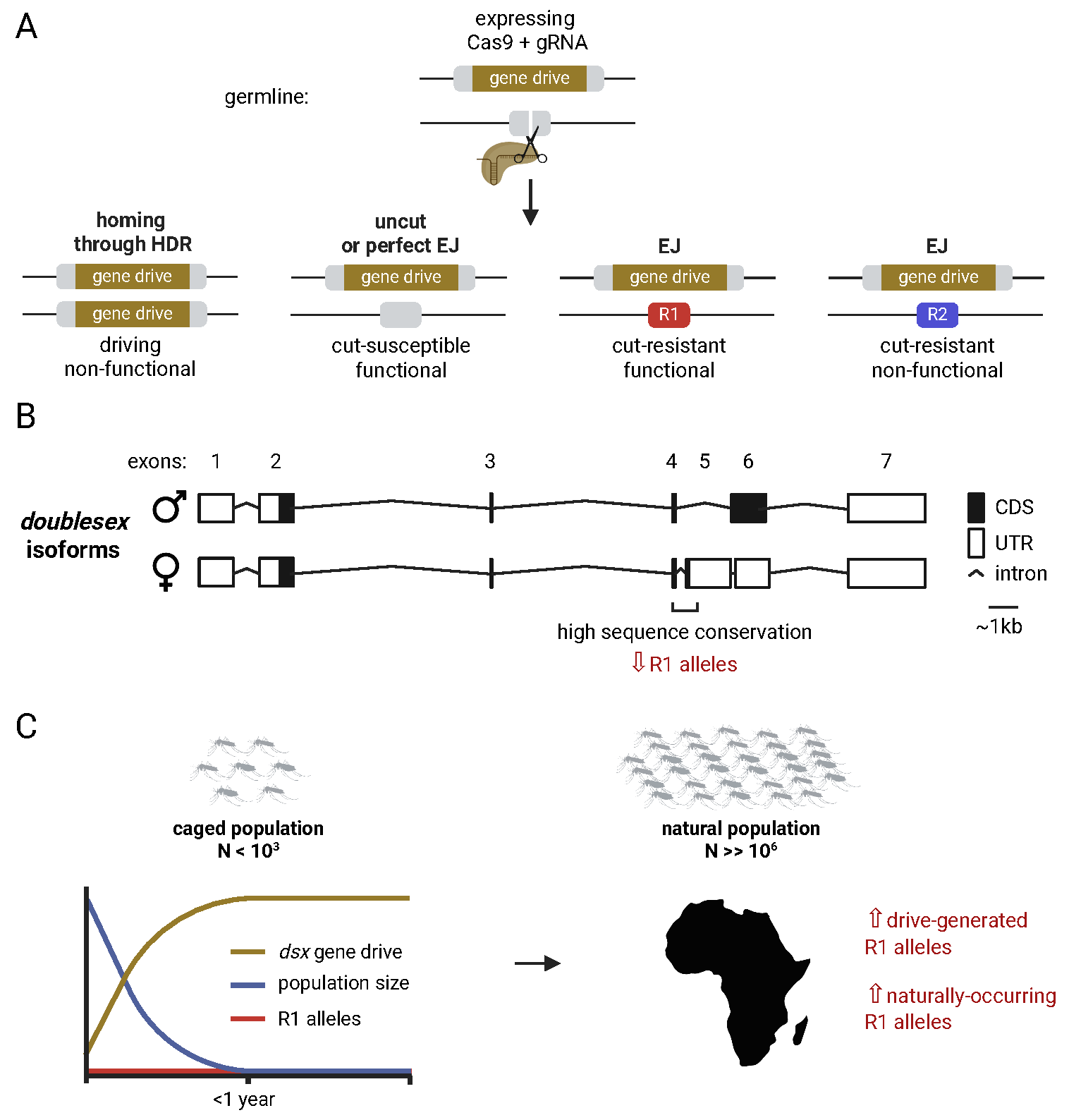

Supplement: S1 Fig — (A) There are four main repair outcomes after a gene drive-derived Cas9 and gRNA catalyze cleavage of an exposed chromosome, carrying their intact target site, in the germline. The most common outcome is homing of the gene drive (light brown) due to homology-directed repair (HDR). Alternatively, the cut chromosome gets repaired by end-joining (EJ). Perfect EJ repairs the wild-type allele (light gray), which is susceptible to further cleavage. EJ is often error-prone and can lead to the introduction of cut-resistant mutations at the gene drive target site that are either functional (R1, red) or non-functional (R2, blue). Functional resistance (R1) has a selective advantage and can reverse gene drive spread. (B) The doublesex gene is expressed into two sex-specific isoforms. Targeting the highly conserved, and presumably functionally constrained region on the intron-exon boundary of the female-specific exon (exon 5) of dsx, can limit R1 alleles. (C) Indeed, no R1 alleles were detected in caged laboratory populations, leading to complete population elimination in less than a year [7,9]. However, natural populations are larger, by several orders of magnitude, which increases the likelihood of drive-induced R1 allele formation, whilst R1 alleles might also be pre-existing in nature. The figure was created in BioRender. Morianou, I. (2026) https://BioRender.com/12gfa3p. (TIF) [file pbio.3003879.s001.tif]

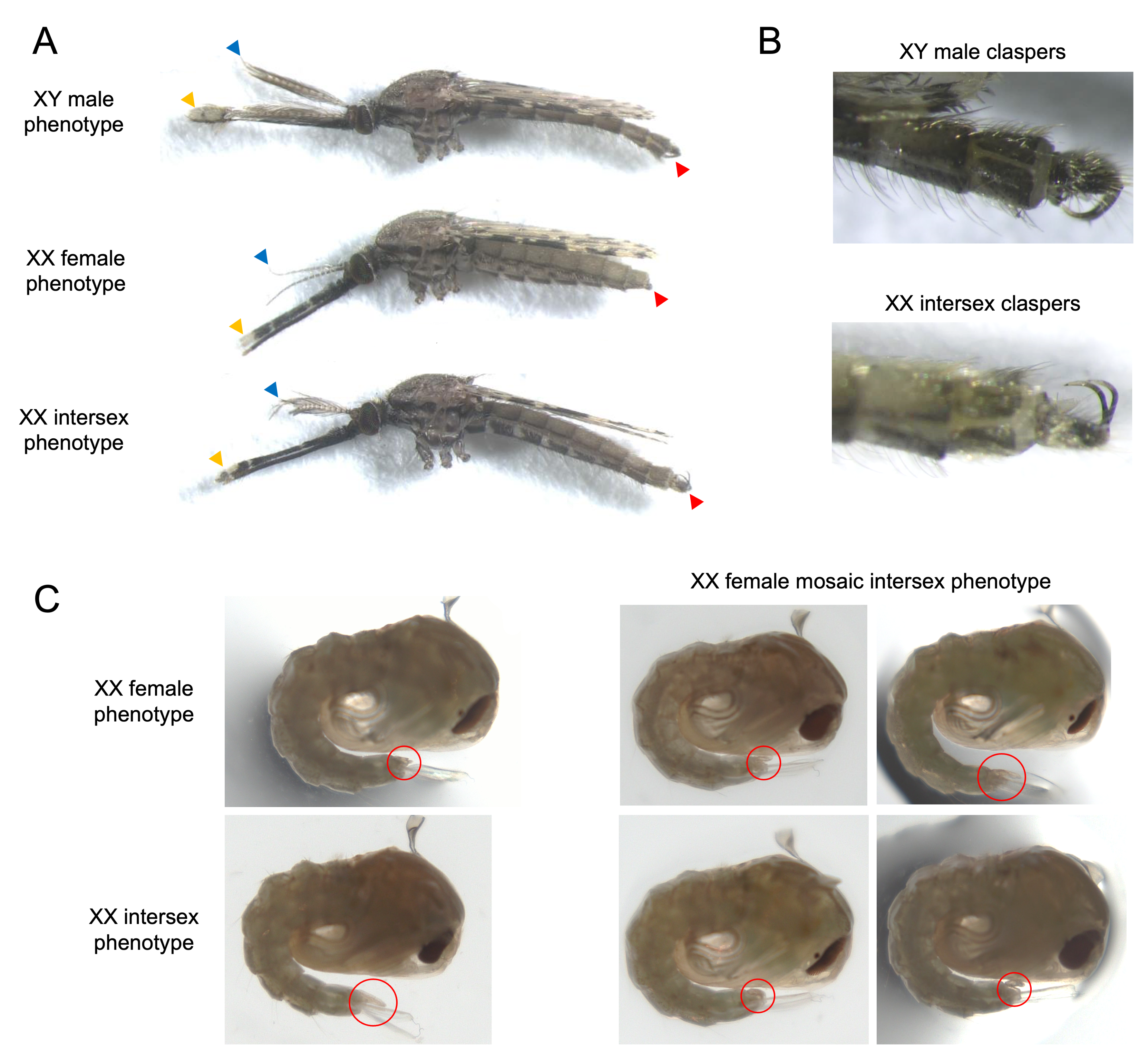

Supplement: S6 Fig — (A) Intersex females develop semi-plumose antennae (blue arrows), resembling that of males; male-like palps, and a proboscis that is unable to draw blood (yellow arrows); and under-developed claspers, which females completely lack, facing upwards, instead of downwards like in males (red arrows) [7]. (B) Claspers rotate to face downwards in mature males, whereas intersex claspers remain facing upright. (C) Pupal genitalia are denoted using red circles. Fully intersex females develop male-like genitalia (uniform phenotype). Mosaic intersex individuals can be distinguished by their under-developed male-like genitalia (variable phenotype, showing different degrees of penetrance depending on the level of mosaicism). (TIF) [file pbio.3003879.s006.tif]

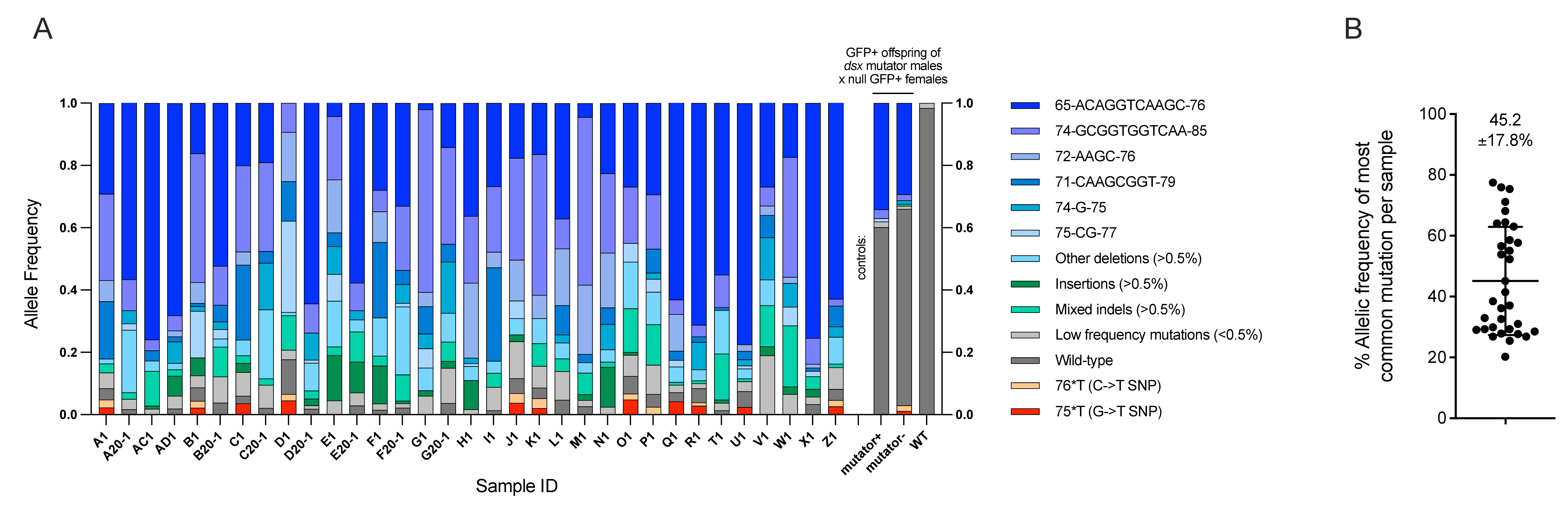

Supplement: S9 Fig — (A) A minimum of 50 mosaic males containing a multitude of Cas9-induced EJ mutations were crossed to a minimum of 50 dsx null (dsxF−)-carrying females (GFP+) [7]. This cross was performed in replicate cages 33 times (cages A, B, C, D, E, F, G, H, I, J, K, L, M, N, O, P, Q, R, T, U, V, W, X, Z, AC, AD, A20, B20, C20, D20, E20, F20, G20). For each cross, 100 GFP+ intersex offspring were analyzed through pooled amplicon Illumina sequencing. The bars show the relative portion of each mutation recovered in intersex individuals (A). The graph shows the allelic frequency of the most common mutation present in each intersex offspring pool (B). As controls, three pools of 100 non-sex-separated individuals were subjected to pooled amplicon Illumina sequencing. The first two pools contained the GFP+ offspring of a cross of 50 dsx mutator males (expressing both a zpg-Cas9 and a gRNA against the target site of Ag(QFS)1) to 50 null GFP+ females, in the absence of the maternal vas2::Cas9 strain. Offspring that had also inherited the dsx mutator allele (mutator+, ALEDL), in addition to the dsxF− GFP+ null allele, were analyzed separately from those that did not inherit the dsx mutator allele (mutator−, DL). The third pool only contained wild-type individuals (WT). The data underlying this figure can be found in S2 Data and https://doi.org/10.5281/zenodo.20541932. (TIF) [file pbio.3003879.s009.tif]

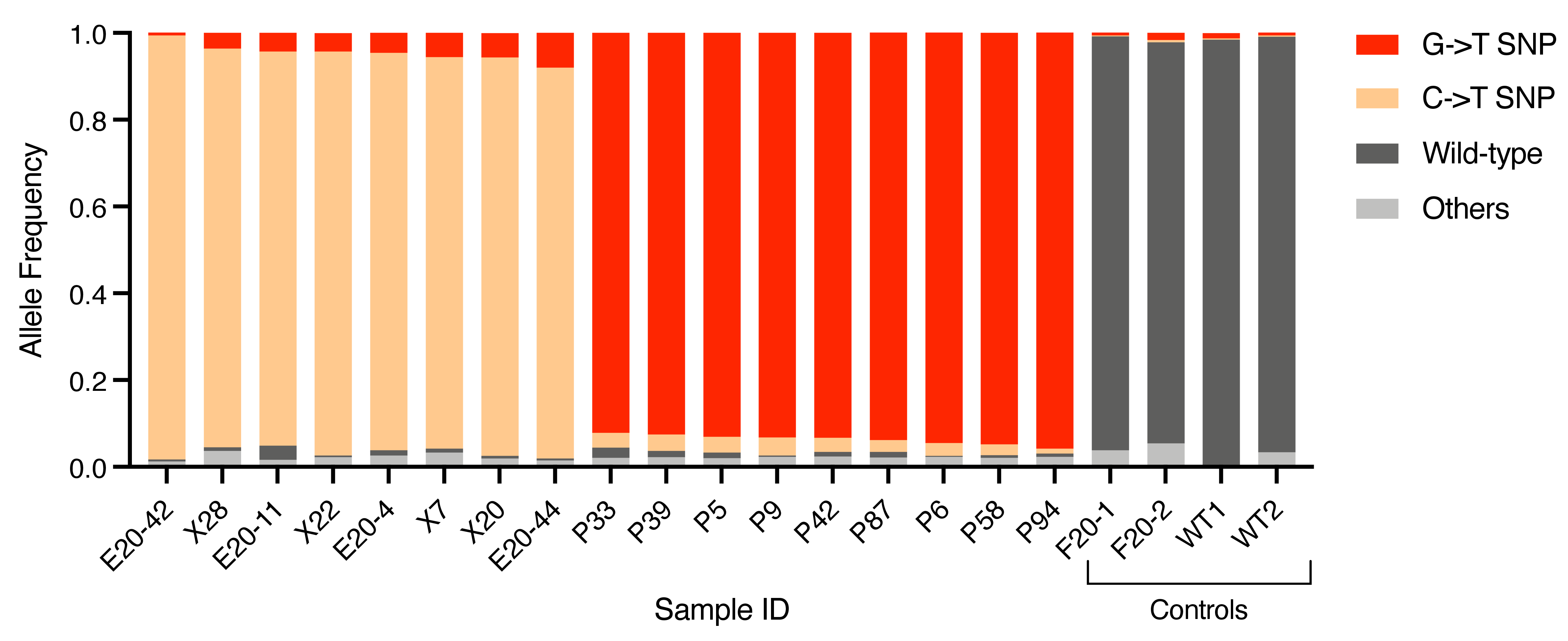

Supplement: S10 Fig — These samples were previously shown to carry a single allele paired to the null dsx mutation through Sanger sequencing: the C→T SNP, designated as R3 (majority light orange), the G→T SNP, designated as R1 (majority red) or a WT allele (majority dark gray). F3 GFP+ individuals were batch-collected from large cages containing >500 mosquitoes and separated on a CO2 pad into three groups comprising of: (1) males, (2) anatomical females, and (3) anatomical intersex, for long-term storage (>6–12 months). Prior to gDNA extraction anatomical females were individually separated. Note that low level of cross-contamination of the samples is possible. The data underlying this figure can be found in S1 Data and https://doi.org/10.5281/zenodo.20545056. (TIF) [file pbio.3003879.s010.tif]

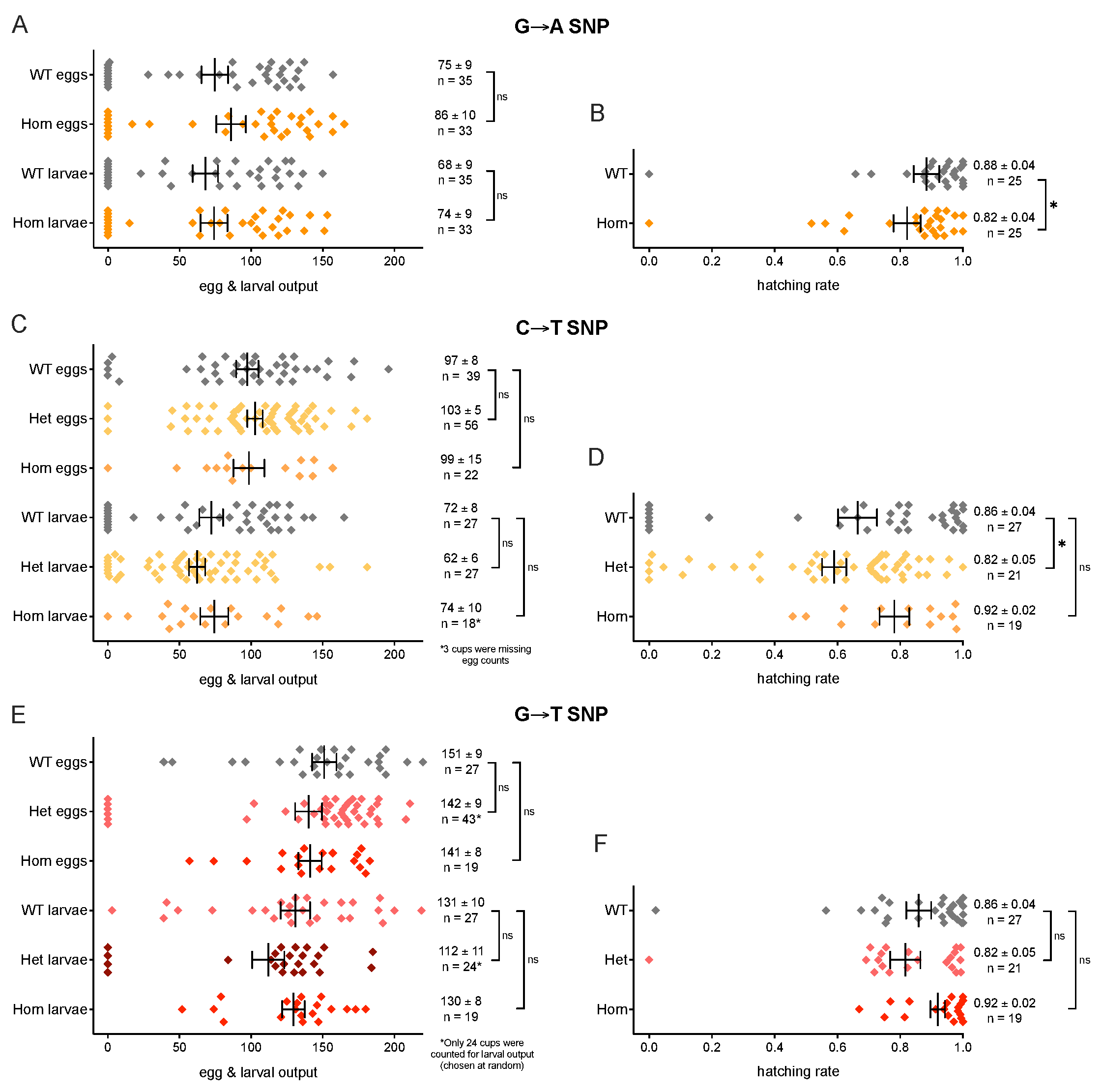

Supplement: S11 Fig — (A) Egg and larval output of females carrying the naturally occurring G→A SNP (non-resistant). The data are not normally distributed (D’Agostino–Pearson normality test). Mann–Whitney non-parametric test: ns: not significant (with p-value = 0.3051, HL diff. = 7.00, 95% CI: −6.00 to 31.00 for Hom vs. WT egg output; and p-value = 0.6459, HL diff. = 0.00, 95% CI: −14.00 to 29.00 for Hom vs. WT larval output). (B) Egg hatching rate of the offspring of females carrying the naturally occurring G→A SNP. The data are not normally distributed (D’Agostino–Pearson normality test). Mann–Whitney non-parametric test: *: significant with p-value = 0.0463, HL diff. = −0.04, 95% CI: −0.09 to 0.00. (C) Egg and larval output of females carrying the Cas9-induced C→T SNP (partially resistant, R3). Only egg output data are normally distributed (D’Agostino–Pearson normality test) and were therefore analyzed using an ordinary ANOVA: ns: not significant, with p-value = 0.7869, mean diff. = −5.41, 95% CI: −25.97 to 15.15 for Het vs. WT egg output, and p-value = 0.9946, mean diff. = −1.17, 95% CI: −31.12 to 28.77 for Hom vs. WT egg output. Larval output data were analyzed using the Kruskall–Wallis non-parametric test and Dunn’s post-hoc test for multiple comparisons: ns: not significant with p-value = 0.4326, 95% CI: −25.37 to 8.50 for Het vs. WT larval output, and p-value > 0.9999, 95% CI: −22.49 to 23.78 for Hom vs. WT larval output. (D) Egg hatching rate of the offspring of females carrying the Cas9-induced C→T SNP. The data are not normally distributed (D’Agostino–Pearson normality test) and were analyzed using the Kruskall–Wallis non-parametric test, and Dunn’s post-hoc test for multiple comparisons: *: significant with p-value = 0.0445, 95% CI: −32.26 to 2.94; ns: not significant with p-value > 0.9999, 95% CI: −20.82 to 30.33. (E) Fertility of females carrying the Cas9-induced G→T SNP (fully resistant, R1). The data are not normally distributed (D’Agostino–Pearson normality test) and wer [file pbio.3003879.s011.tif]

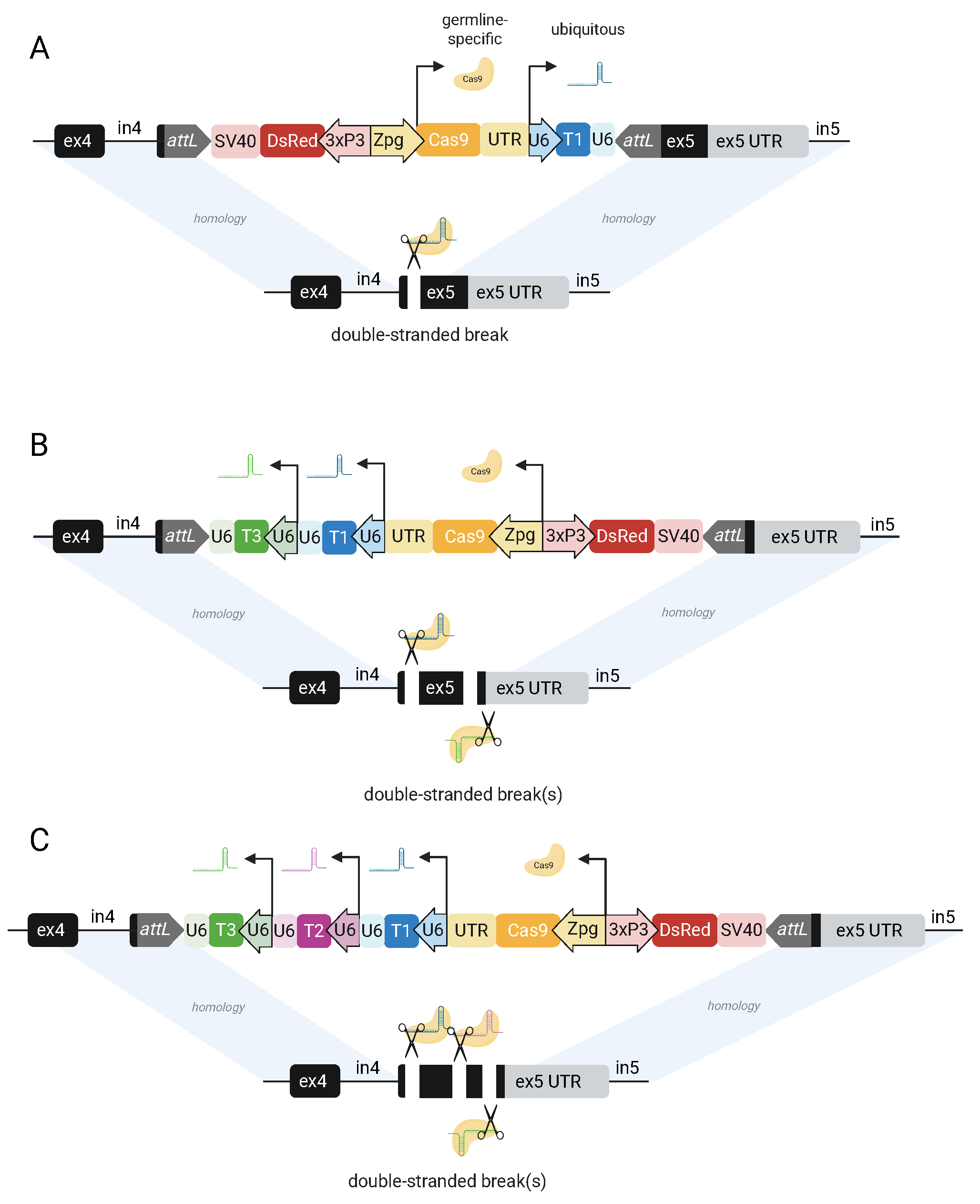

Supplement: S12 Fig — (A) The Ag(QFS)1 gene drive construct integrated in the same orientation as the doublesex gene. (B) The Ag(QFS)2 gene drive construct integrated in the reverse orientation with respect to dsx. (C) The Ag(QFS)3 gene drive construct integrated in the reverse orientation with respect to dsx. Gene drive components: att: = RMCE ruminant attachment sites, SV40 = viral terminator, RFP = DsRed fluorescent marker, 3xP3 = neuronal promoter, zpg = zero population growth promoter and untranslated region (UTR), Cas9 = human codon-optimized Streptococcus pyogenes Cas9 (SpCas9) gene, U6 = pol III promoter and terminator, T1/2/3 = gRNAs containing spacer sequences complementary to sites T1, T2 or T3. The figure was created in BioRender. Morianou, I. (2026) https://BioRender.com/p42nzim. (TIF) [file pbio.3003879.s012.tif]

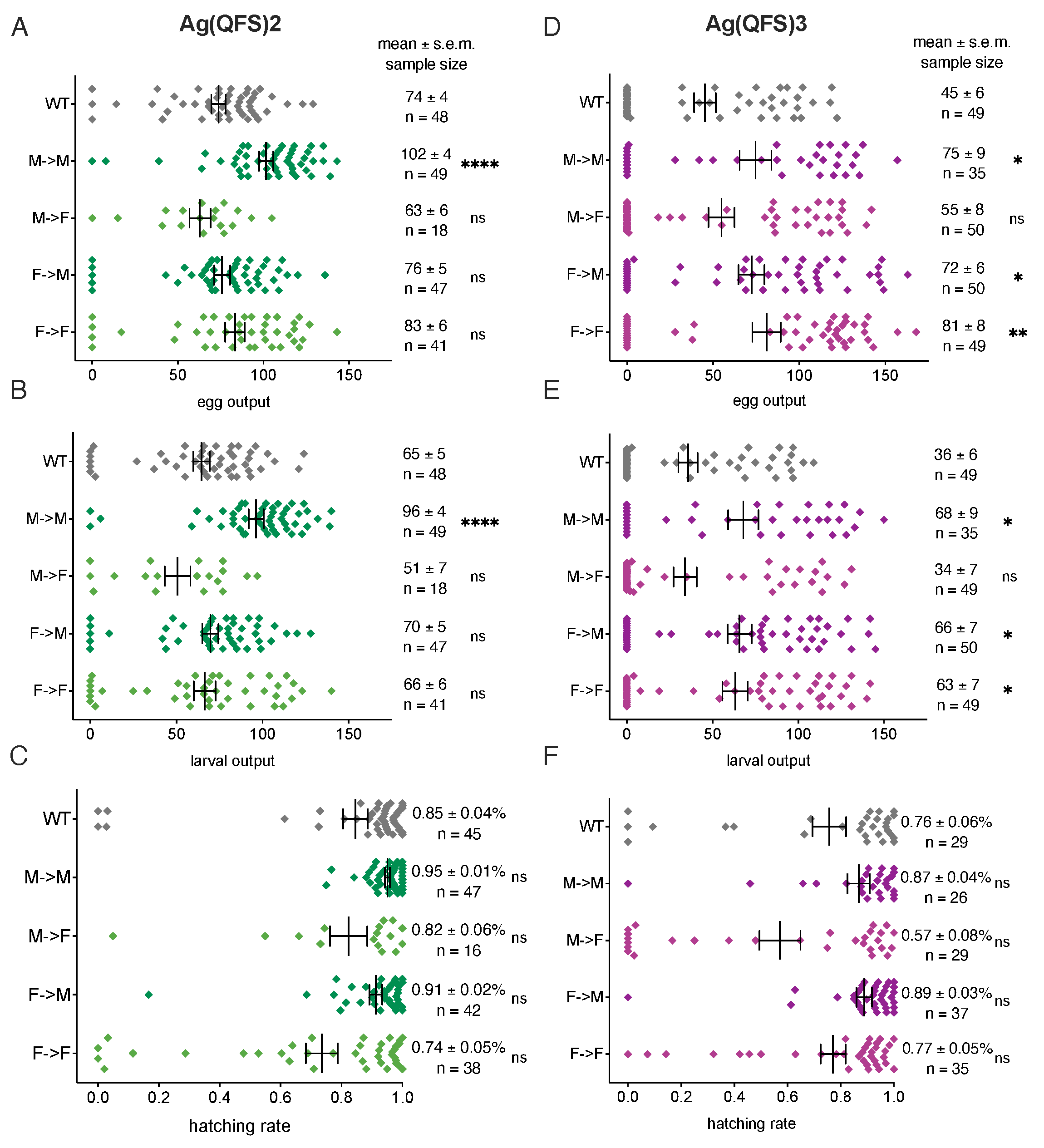

Supplement: S13 Fig — Male (M) and female (F) heterozygous gene drive carriers that inherited the paternally (M→M, M→F) or maternally (F→M, F→F) were crossed to wild-type, and the number of eggs (A, D) and larvae (B, E) produced per female parent were scored. The hatching rate of the eggs is also shown (C, F). Only mated females are included in the Ag(QFS)2 analysis (A–C) and both mated and unmated females are included in the Ag(QFS)3 analysis (D–F). Mean values, the standard error around the mean (S.E.M.), together with each sample size (n) are shown to the right of each graph, for each cross. The fertility of Ag(QFS)1 and Ag(QFS)3 gene drive carriers was compared to the wild-type control using a Kruskall–Wallis non-parametric test with Dunn’s post-hoc multiple comparisons test, since the data were not normally distributed (D’Agostino–Pearson). The data underlying this Figure can be found in S8 Data, and the corresponding statistical analyses in S5 Data. (TIF) [file pbio.3003879.s013.tif]

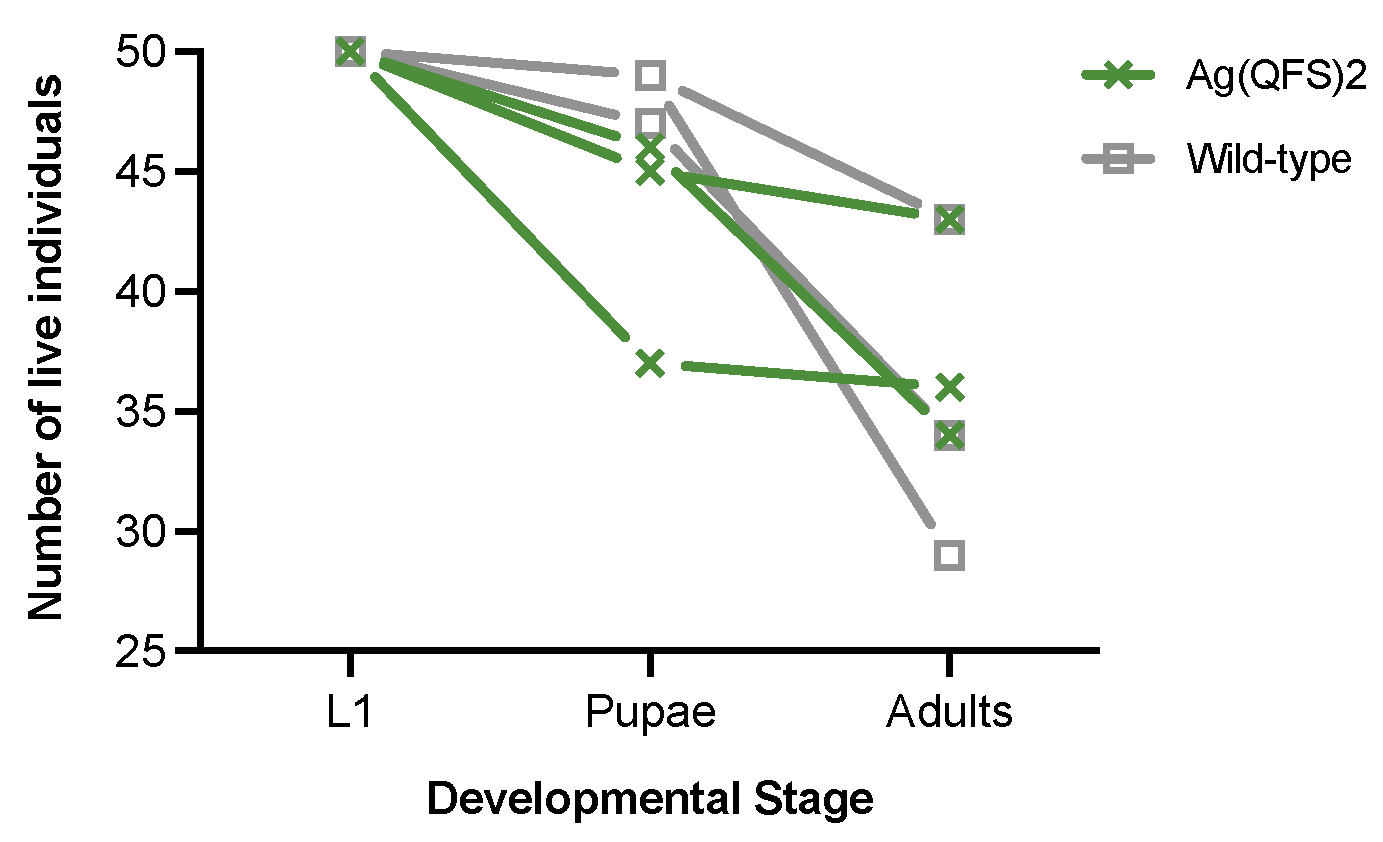

Supplement: S14 Fig — Starting from 50 Ag(QFS)2 and 50 wild-type L1 larvae per tray per triplicate, the number of individuals that survived into becoming pupae and adults were recorded. On average, 85.3% of Ag(QFS)2 L1 larvae pupated, compared to 96.7% of wild-type L1 larvae; and 75.3% of Ag(QFS)2 L1 larvae reached adulthood, compared to 70.7% of wild-type L1 larvae. The data passed the Shapiro–Wilk test for normality and were analyzed using a mixed effects two-way ANOVA, taking into account the paired nature of the data, comparing the Ag(QFS)2 (green) and wild-type (gray) groups across developmental stages (L1→pupae→adults), to find no significant difference between them (F(1.476, 5.905) = 1.653, p-value = 0.2611). The data underlying this Figure can be found in S8 Data, and the corresponding statistical analyses in S5 Data. (TIF) [file pbio.3003879.s014.tif]

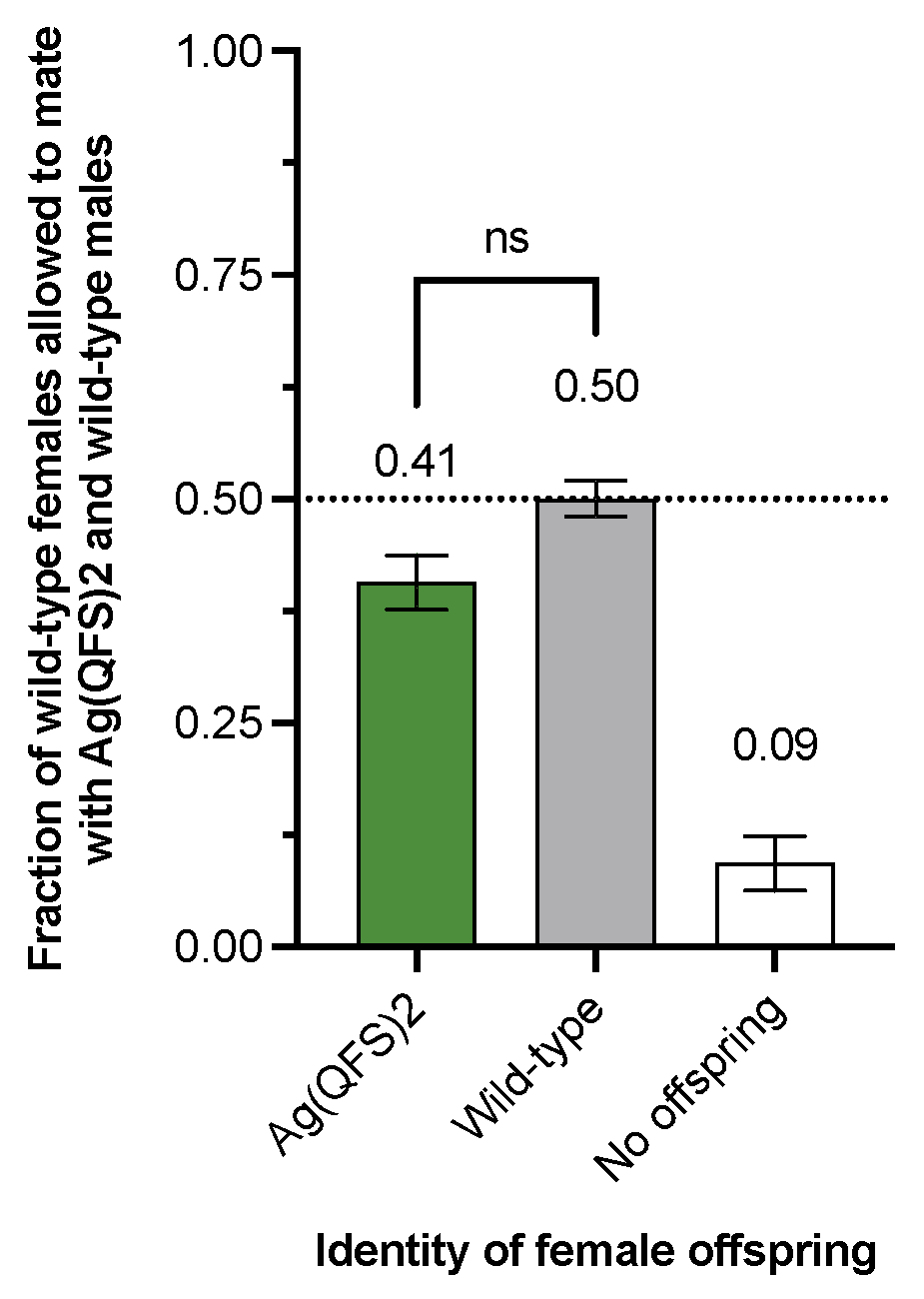

Supplement: S15 Fig — Fifty Ag(QFS)2 heterozygous males competed against 50 wild-type males for mating with 60 wild-type females, in triplicate. The identity (Ag(QFS)2 in green or wild-type in gray) of the offspring of 50 randomly selected females per triplicate revealed whether those females had mated with Ag(QFS)2 carriers or wild-type males. On average (mean ± SD), a greater fraction of females had mated with wild-type (0.50 ± 0.02) than Ag(QFS)2 males (0.41 ± 0.03), however this difference was not significant as deduced by paired t test (p-value = 0.0604; 95% CI: −0.010 to 0.197, ηp2 = 0.8829), after the data passed the Shapiro–Wilk test for normality. For clarity the fraction of females that did not produce successful matings (0.09 ± 0.03) were also plotted. Means are shown above each column and error bars represent the standard deviation (SD). The data underlying this Figure can be found in S8 Data, and the corresponding statistical analyses in S5 Data. (TIF) [file pbio.3003879.s015.tif]

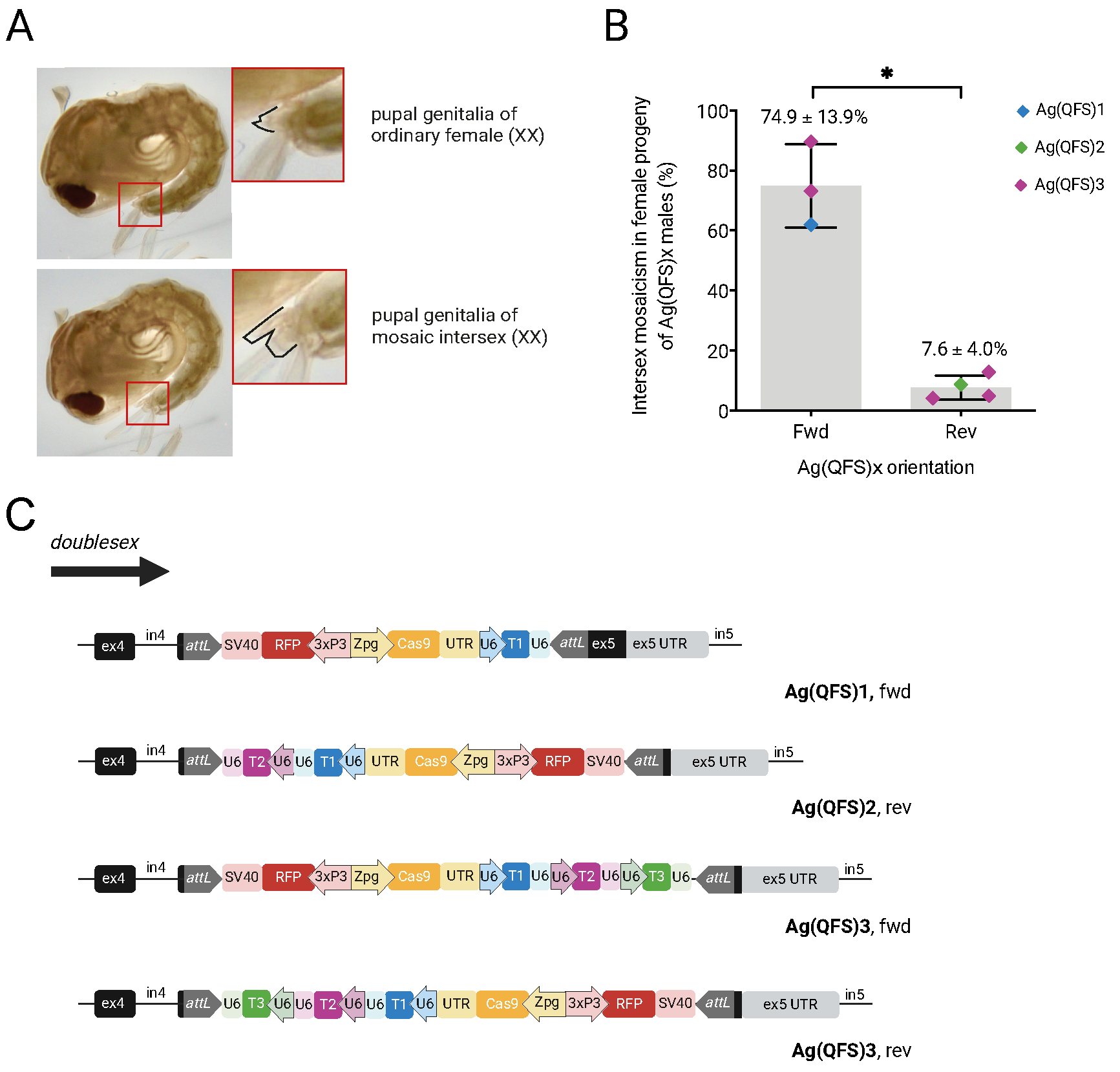

Supplement: S17 Fig — (A) Example of the female genitalia at the pupal stage, of phenotypic females (top) versus mosaic intersex females (bottom). (B) Percentage of intersex mosaics in the female offspring of male gene drive carriers of Ag(QFS)1, Ag(QFS)2 or Ag(QFS)3 that harbored the gene drive in the same (fwd) or reverse (rev) orientation in the genome, with respect to dsx. Means and standard deviations are shown above the graph and indicated by the error bars. A t test with Welch’s correction to account for unequal standard deviation in the two datasets was performed with p-value = 0.0104, η2 = 0.9671, 95% CI: −99.34 to −35.20. Note that each data point originated from an independently generated strain (i.e., isolated from a different founder). (C) Illustrations of the gene drive integration mode with respect to dsx (fwd or rev) for each of the gene drives examined. The data underlying Panel B can be found in S8 Data, and the corresponding statistical analyses in S5 Data. Panel C was created in BioRender. Morianou, I. (2026) https://BioRender.com/q7ssenl. (TIF) [file pbio.3003879.s017.tif]
