# Supplementary material for: Engineering resilient gene drives for sustainable malaria control by predicting, testing and overcoming target site resistance in Anopheles gambiae
Source: PLoS Biol. 2026 Jul 6;24(7):e3003879. doi: 10.1371/journal.pbio.3003879 (PMC13395382; doi:10.1371/journal.pbio.3003879)
Supplement: S1 Table — (DOCX) [file pbio.3003879.s019.docx]

| **GFP^+^ genetic females collected** | | **N** | **%** |
| --- | --- | --- | --- |
| Total | | 10,083 | 100.0 |
| Female anatomy | | 1,031 | 10.2 |
| Intersex anatomy | | 9,052 | 89.8 |
|  | |  |  |
| **Sanger-sequenced anatomical females** | | **N** | **%** |
| Total |  | 852 | 100.0 |
|  | Blood-fed | 629 | 94.8 |
| Wild-type | Non blood-fed | 107 |  |
|  | Unchallenged | 72 |  |
| C->T SNP | Blood-fed | 14 | 2.2 |
|  | Non blood-fed | 5 |  |
| G->T SNP | Blood-fed | 9 | 1.1 |
|  | Non blood-fed | 0 |  |
| Others modified | Blood-fed | 2 | 0.4 |
|  | Non blood-fed | 1 |  |
| Mixed modified alleles* | Blood-fed | 10 | 1.5 |
|  | Non blood-fed | 3 |  |

*****Likely resulting from mosaicism due to residual *zpg-*expressed Cas9 activity.
