# Supplementary material for: Engineering resilient gene drives for sustainable malaria control by predicting, testing and overcoming target site resistance in Anopheles gambiae
Source: PLoS Biol. 2026 Jul 6;24(7):e3003879. doi: 10.1371/journal.pbio.3003879 (PMC13395382; doi:10.1371/journal.pbio.3003879)
Supplement: S2 Table — See also S18 Fig for an illustration of how the modeling parameters relate to each other. (DOCX) [file pbio.3003879.s020.docx]

| **Parameters** | **Description** | **Values** |
| --- | --- | --- |
| $\varepsilon_{W}$ | Cleavage rate of WT that leads to non-WT allele | 0.966 |
| ${1-\varepsilon}_{W}$ | Frequency of unmodified WT allele after exposure to nuclease | 0.034 |
| $\nu$ | Frequency of WT allele conversion to EJ mutant allele | 0.035 |
| $1-\nu$ | Fraction of drive alleles amongst those cleaved | 0.965 |
| $\beta$ | Fraction of EJ mutations that become an R1 allele | 0.0016 |
| $1-\beta-\gamma$ | Fraction of EJ mutations that become an R2 allele | 0.996 |
| $\gamma$ | Fraction of EJ mutations that become an R3 allele | 0.0025 |
| $\varepsilon_{R3}$ | Cleavage rate of R3 alleles | 0.416 |
| ${1-\varepsilon}_{R3}$ | Frequency of unmodified R3 alleles after exposure to nuclease | 0.584 |
| $\nu$’ | Frequency of R3 conversion to EJ mutant allele | 0.000 |
| $1-\nu$’ | Fraction of drive alleles amongst those cleaved | 1.000 |
| $\beta'$ | Fraction of EJ mutations that become an R1 allele | 0.000 |
| $1-\beta'-\gamma'$ | Fraction of EJ mutations that become an R2 allele | 1.000 |
| $\gamma$’ | Fraction of EJ mutations that become a WT allele | 0.000 |
| - | Fraction of non-drive, cleaved WT alleles that are converted to EJ mutant alleles | 0.500 |
| - | Fraction of non-drive, cleaved R3 alleles that are converted to EJ mutant alleles | 0.020 |
